# Halo(natrono)archaea isolated from hypersaline lakes utilize cellulose and chitin as growth substrates

**DOI:** 10.3389/fmicb.2015.00942

**Published:** 2015-09-15

**Authors:** Dimitry Y. Sorokin, Stepan V. Toshchakov, Tatyana V. Kolganova, Ilya V. Kublanov

**Affiliations:** ^1^Winogradsky Institute of Microbiology, Research Centre of Biotechnology, Russian Academy of SciencesMoscow, Russia; ^2^Department of Biotechnology, Delft University of TechnologyDelft, Netherlands; ^3^Immanuel Kant Baltic Federal UniversityKaliningrad, Russia; ^4^Institute of Bioengineering, Research Centre of Biotechnology, Russian Academy of SciencesMoscow, Russia

**Keywords:** halo(natrono)archaea, hypersaline lakes, soda lakes, cellulose, chitin, cellulotrophic, chininotrophic

## Abstract

Until recently, extremely halophilic euryarchaeota were considered mostly as aerobic heterotrophs utilizing simple organic compounds as growth substrates. Almost nothing is known on the ability of these prokaryotes to utilize complex polysaccharides, such as cellulose, xylan, and chitin. Although few haloarchaeal cellulases and chitinases were recently characterized, the analysis of currently available haloarchaeal genomes deciphered numerous genes-encoding glycosidases of various families including endoglucanases and chitinases. However, all these haloarchaea were isolated and cultivated on simple substrates and their ability to grow on polysaccharides *in situ* or *in vitro* is unknown. This study examines several halo(natrono)archaeal strains from geographically distant hypersaline lakes for the ability to grow on insoluble polymers as a sole growth substrate in salt-saturated mineral media. Some of them belonged to known taxa, while other represented novel phylogenetic lineages within the class *Halobacteria*. All isolates produced extracellular extremely salt-tolerant cellulases or chitinases, either cell-free or cell-bound. Obtained results demonstrate a presence of diverse populations of haloarchaeal cellulo/chitinotrophs in hypersaline habitats indicating that euryarchaea participate in aerobic mineralization of recalcitrant organic polymers in salt-saturated environments.

## Introduction

Hypersaline lakes and salterns with salt concentrations close to saturation (normally above 200 g l^-1^) represent border-of-life habitats with extremely salt-tolerant prokaryotic communities. In general, the brines are a domain of extremely halophilic euryarchaea belonging to the class *Halobacteria*, while extremely halophilic bacteria dominate the sediments. According to the current knowledge, haloarchaea are mainly represented by aerobic heterotrophs, with few exceptions represented by facultative anaerobes capable of utilizing simple soluble organic monomers (Horikoshi, 2011; Ventosa et al., 2011; Andrei et al., 2012; Oren, 2013). Haloarchaea typically have very high cell density that gives the characteristic reddish color to hypersaline brines. This high density of haloarchaea was attributed to evaporative concentration not only of inorganic but also of organic molecules. Some haloarchaea are capable of hydrolyzing polymeric substances, such as starch, proteins, and olive oil (Bhatnagar et al., 2005; Enache and Kamekura, 2010; Moshfegh et al., 2013; Selim et al., 2014); however, this ability and potential participation of haloarchaea in mineralization of insoluble organic polymers have not been studied systematically and this function in hypersaline habitats was attributed to halophilic bacteria exclusively (Andrei et al., 2012; Oren, 2013).

The ability of haloarchaea to hydrolyze recalcitrant polysaccharides caused significant interest recently because hydrolases that catalyze this process should be extremely salt-tolerant. One particular application of such enzymes is in biofuel production from lignocellulosic wastes because this process includes a de-crystallizing pre-treatment step, either with alkali or ionic liquids (Kaar and Holtzapple, 2000; Zavrel et al., 2010; Begemann et al., 2011).

Although genes encoding putative cellulases were found in many haloarchaeal genomes (Supplementary Table S1), the presence of functional cellulases have been demonstrated only for two genera – *Halorhabdus* and *Haloarcula*. The *Halorhabdus utahensis* genome contains a gene cluster that encodes several glycosidases (GHs) of GH5 and GH9 families. One GH5 cellulase was cloned and studied biochemically. This enzyme is an endoglucanase, which is not only salt-tolerant, but also thermo- and alkali-stable. The enzyme, however, was not tested for the ability to hydrolyze insoluble celluloses likely because this haloarchaeaon cannot grow on these substrates (Zhang et al., 2011). Another study has characterized two phylogenetically nearly identical strains of *Haloarcula* obtained from saline soils in China (Li and Yu, 2013a,b). Both strains produced endoglucanases that hydrolysed the soluble cellulose analogue carboxymethylcellulose (CMC) at salt concentrations up to saturation. One strain produced a single endoglucanase, whereas the second strain secreted five endoglucanases of different molecular weights. The cellulase cocktail from the latter strain released reducing sugars from alkali-pretreated rice straw at extreme salinity. This study, however, similar to study of Zhang et al. (2011), did not examine whether these two cellulolytic haloarchaea would grow on insoluble celluloses.

The genes encoding GH18 family chitinases were found in the genomes of several haloarchaea (Supplementary Table S1). Two of them (*Halobacterium salinarum* and *Haloferax mediterranei*) produce functionally active chitinases. An earlier study of ChiN1 enzyme cloned from *Hbt. salinarum* NRC-1 identified a single three-domain structure typical for the GH18 chitinases, and demonstrated that this secreted protein can degrade insoluble chitin at salt-saturating conditions; yet, surprisingly, optimal enzymatic activity was observed at a relatively low salinity (Hatori et al., 2005, 2006; Yatsunami et al., 2010). However, the ability of the strain to grow with chitin as substrate has not been tested. In contrast, a recent work on chitin degradation by *Hfx. mediterranei* for the first time demonstrated that this haloarchaeaon can grow on insoluble chitin producing four cell-bound chitinases encoded by a single operon (Hou et al., 2014). Still, the work was initially based on genomic information and not by enriching an organism from its natural habitat on the basis of a unique functional property.

Yet, it remains unclear whether haloarchaea can act as cellulo- and chitinotrophs in their natural hypersaline habitats. Our study addresses this question and shows that many haloarchaea obtained from brines and sediments of geographically distant hypersaline lakes can be specifically enriched and isolated on and use natural insoluble forms of cellulose and chitin as sole growth substrates, thus playing a role of primary destructors in their environments.

## Materials and Methods

### Samples

Sediment (first 10 cm) and brine samples were obtained from hypersaline chloride-sulfate lakes and salterns located in Central Asia, southern Russia, Crimea, and Spain and from hypersaline alkaline and soda lakes in south-west Siberia (Supplementary Table S2).

### Enrichment and Growth Conditions

Neutrophilic haloarchaea were enriched and maintained on medium A containing (in g l^-1^) 240 NaCl, 2 KCl, 0.25 NH_4_Cl, and 2.5 of K_2_HPO_4_/KH_2_PO_4_, pH 6.8. After sterilization, the base components were supplemented with vitamin and trace metal mix (Pfennig and Lippert, 1966), 1 mM MgSO_4_, 10 mg l^-1^ yeast extract and 10 mM filter-sterilized NaHCO_3_. Haloalkaliphilic archaea from soda lakes were enriched and cultivated on sodium carbonate-based medium containing 4 M total Na^+^ mixed in 1:1 proportion with medium A. The sodium carbonate base included (in g l^-1^) 185 Na_2_CO_3_, 45 NaHCO_3_, 16 NaCl, and 1 K_2_HPO_4_ with pH 10–10.1 after sterilization. Same ingredients were added after sterilization as for NaCl medium and resultant solution was supplemented with sterile 4 mM NH_4_Cl to prepare medium B. After 1:1 mixing, pH of the resulting medium C was adjusted to 9.6. Various forms of insoluble celluloses with different degrees of crystallinity and crystalline chitin from crab shell (Sigma) were used as growth substrates at the final concentration of 1–2 g l^-1^. Amorphous (colloidal) cellulose and chitin were prepared by dissolving crystalline polymers in concentrated H_3_PO_4_ on ice and subsequent dilution in large volumes of ice-cold distilled water with subsequent neutralization and concentration by low-speed centrifugation. The final suspension was adjusted to 5% (w/v) concentration and sterilized at 120°C for 20 min in closed bottles. Routine cultivation was performed in 20 ml liquid medium in 120 ml serum bottles tightly closed with grey-rubber septa at 37°C and shaking at 150 rpm. Solid media were prepared by 1:1 mixing of the fully prepared liquid media A or B with 4% agarose at 48°C. Before mixing, the liquid media were supplemented with solid NaCl to compensate for dilution with agarose and to bring the salinity to 4 M of total Na^+^. The use of amorphous cellulose or chitin in solid media enabled discrimination of colonies of hydrolytic haloarchaea from those without hydrolytic activity because hydrolytically active colonies formed a hydrolysis clearance zone around. Isolation of pure cultures was achieved by combining iterative limiting dilutions with colony purifications, because dilutions alone did not exclude growth of contaminating non-hydrolytic haloarchaea fed by scavenging the soluble hydrolysis products.

To investigate the effect of salinity on growth and hydrolytic activity in culture fractions, medium A was prepared with NaCl concentrations varying from 1 to 5 M, while in medium B total Na^+^ content varied from 1 to 4 M. Alkaline media with variable Na^+^ concentrations (from 1 to 4.8 M) were prepared by mixing medium B with medium A (in most cases at 1:1 proportions) and adjusting total Na^+^ by titration with 4 M NaOH to bring the final pH to 9.6, if required. The pH dependence was studied in media containing 4 M total Na^+^ using following buffers:0.1 M K-phosphate buffer for the pH range 6–8; 0.05 M K-P/0.1 M NaHCO_3_ for pH 8–8.5; NaHCO_3_/Na_2_CO_3_ for pH 9–11.

### Measurements of Hydrolytic Activity

Hydrolytic activity was measured in culture supernatant concentrated 10 times using 10 kDa-pore protein concentrators (Millipore) or in cell extracts obtained by sonication of cell pellets. Hydrolytic activity at different pH and salinity was quantified by agar-diffusion technique on plates using either amorphous polymers or CMC or soluble xylanes, at 0.1% concentration each. Before measurements, the plates were soaked with distilled water for several hours with several changes to reduce the background salt concentration in the agarose plates. Hydrolysis of amorphous polymers was detected by monitoring the clearance zones formed around the wells, while endoglucanase and endoxylanase activity were visualized by staining the plates with 0.1% Congo Rod (Sigma) for 30 min followed by 1 M NaCl wash. Hydrolytic activity was also quantified by analyzing reducing sugar release during incubations of 2 ml fractions in 7 ml closed serum bottles with 1 g l^-1^ polymers at 37°C and shaking at 100 rpm.

### Analytical Methods

The production of reducing sugars was measured in supernatants by the nitrosalicylate method as described elsewhere (Miller, 1959). Hydrolytic activity was calibrated with glucose or *N*-acetyglucosamine for cellulose and chitinase activity measurements, respectively. Biomass was quantified by measurements of total cell protein because isolates attach firmly to the insoluble polymeric phase. The cultures were first homogenized by vigorous shaking and 1–2 ml aliquots centrifuged. The pellets were resuspended in 0.5 ml distilled water. These manipulations caused lysis of free and attached haloarchaeal cells. The samples were stored at -20°C until further analysis. After thawing, 0.5 ml 2 M NaOH was added to the suspension. After 30 min incubation at room temperature, the remaining solids were removed by centrifugation. The solubilized protein content was determined by Bradford method (Bradford, 1976). Uninoculated media served as controls. The growth experiments were conducted in triplicates, and the average values were used for analysis. The PAAG electrophoresis and zymography for the β-1,4 endoglucanase activity measurements were performed as described previously (Li and Yu, 2013a). Phase contrast microphotographs were obtained using a Zeiss Axioplan Imaging 2 microscope (Göttingen, Germany). In some cases, cells colonizing cellulosic fibers were stained with green fluorescent nucleic acid dye SYTO-9 (Invitrogen kit L7012) according to a standard protocol to improve images.

### Phylogenetic Analysis

Genomic DNA from pure cultures was extracted and purified using a microbial DNA isolation kit (MoBio). The 16S rRNA genes were amplified using arch8f-1492r primers. In several strains, direct sequencing has determined the presence of several 16S rRNA genes, which is common for several *Halobacteria* (Cui et al., 2009). In such cases the PCR products were cloned using the pGEM-T vector system (Promega) and *Escherichia coli* DH10b competent cells according to the manufacture’s protocol and 20 clones sequenced. In majority of cases the almost complete (>1300 bp) 16S rRNA gene sequences were obtained, however, the lengths of 7 sequences (HArcel3, AArcel1, AArcel6, AArcel4, HArcht-Cr operon 1, HArcht-Cr operon 2, and AArcht6) were in the range 360–880 bp. The long and short sequences were combined in two different sets. To construct a reference database, all haloarchaeal sequences of ARB-SILVA (Quast et al., 2013) SSU 121 database (Archaea/Euryarchaeota/*Halobacteria*) were obtained. Among almost 12000 sequences, 521 representing type species were selected. The sequences were filtered with 100% identity filter using CD-HIT suite (h-cd-hit-est option, Huang et al., 2010) resulting in a 388 sequences set. The set was split into long (>1400 bp) and short (<1400 bp) sets, both of which were slightly manually corrected. Long sets of query and reference sequences were combined with *Methanocella paludicola* and *Thermococcus sibiricus* as outgroups and aligned in the Muscle aligner within the Mega 6 software (Tamura et al., 2013) with default parameters. The alignment was used for phylogenetic analysis in Mr. Bayes 3.25 (Huelsenbeck and Ronquist, 2001). Graphical interpretation of the analysis of 14000000 generations resulted in a phylogenetic tree with 956 branches. The tree then was exported to the Newick format using FigTree 1.4.2. The “long” alignment and the tree were imported into ARB 6.02 software (Westram et al., 2011). After all sequences of <1000 bp were eliminated from the short reference sequences set, it was verified on the presence of same species as in the long reference sequence sets and only unique sequences were retained. Short sets of query and reference sequences were combined with degapped long alignment (see above) and aligned using Muscle with default parameters. After degapping, the resulted alignment was imported into the ARB and placed on the previously constructed tree using the ARB-parsimony tool.

### Genbank Accession Numbers

The 16S rRNA gene sequences generated in this study were deposited in the GenBank under accession numbers KT247945-KT247984.

## Results

### Chitinotrophic Haloarchaea in Hypersaline Lakes with Neutral pH

**FIGURE 1:**
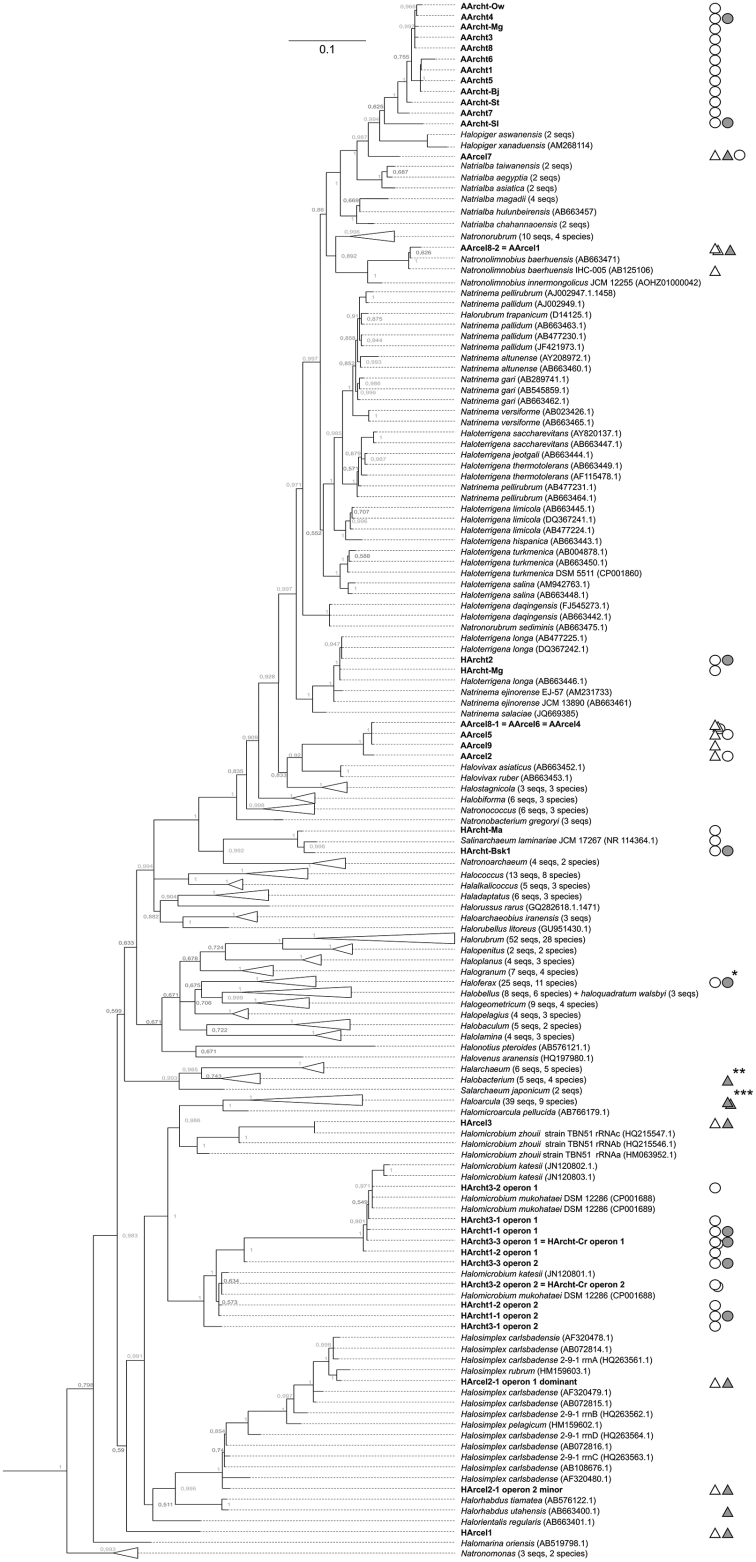
**16S rRNA gene-based Bayesian phylogenetic tree showing the position of chitino/cellulolytic strains among haloarchaea.** The probability that the associated taxa clustered together is shown next to the branches. The numbers at the nodes indicate the bootstrap values. Bar is 10 nucleotide substitutions per 100. The strains growing on chitin are marked by open circles; the strains with characterized chtinolytic activities/chitinases are indicated by solid circles; the strains, growing on insoluble cellulose are indicated by open triangles; the strains with characterized cellulolytic activities/cellulases are indicated by solid triangles. ^∗^*Haloferax mediterranei* ATCC 33500; ^∗∗^*Haloarcula* sp. LLSG7 and *Haloarcula* sp. G10; ^∗∗∗^*Halobacterium salinarum* NRC-1.

Inoculates obtained from sediments or brines of hypersaline chloride-sulfate lakes demonstrated growth in 7 of 8 cultures after 2- to 4-week incubation on medium A supplemented with either powdered crystalline or amorphous chitin as a sole source of energy and carbon (**Table 1**). All positive enrichment cultures developed characteristic pinkish color concurrently with significant degradation of chitin. After 1–2 weeks of incubation of the next (1/100, v/v) transfers the color intensified, synchronously with full degradation of chitin. Addition of bacteria-specific antibiotics (vancomycin, streptomycin, and kanomycin) at concentrations up to 0.5 g l^-1^ did not affect growth and chitin degradation indicating that all enrichments were dominated by haloarchaea. Separation of chitinolytic haloarchaea from the others was achieved by using solid medium A with amorphous chitin as sole substrate; the developed colonies formed visible clearance zones typically in a month. Totally, ten pure cultures of haloarchaea that grow on crystalline or amorphous chitin at 4 M NaCl from samples collected in seven hypersaline lakes have been isolated (**Table 1**). The phylogenetic analysis revealed the isolates belong to three genera of haloarchaea, *Halomicrobium, Haloterrigena/Natrinema*, and *Salinarchaeum* (**Figure 1**).

**Table 1:** Haloarchaeal chitinotrophic strains isolated from hypersaline lakes with neutral pH.

Strain	Source	Morphology		Affiliation	Collection No. in UNIQEM^b^
		Colonies	Cells		
HArcht1-1	Cock salt lake, (S)^a^	Orange-red, semisolid	Motile flat irregular rods and cocci	*Halomicrobium mukohataei*	U963
HArcht1-2	Cock salt lake, (B)^a^	Red, solid	Free cells are motile flat rods, cells aggregated with chitin are irregular flat coccoids		
HArcht3-1	Hummocky salt lake (S)	Red, semisolid	Motile flat rods		U964
HArcht3-2	Lomovoe salt lake (S)	Red, soft	Motile flat rods		
HArcht3-3	Lomovoe salt lake (B)	Red, semisolid	Motile flat rods		
HArcht2	Cock salt lake, (S)	Pink, flat, skinny	Flat irregular cocci and rods	*Haloterrigena longa*	U965
HArcht-Mg	Barun Davst Nur (S+B)				
HArcht-Cr	Crimean salt lakes (S+B)	Red, soft	Motile flat rods	*Halomicrobium mukohataei*	
HArcht-Bsk	Lake Baskunchak (S+B)	Pale pink, soft	Motile flat rods	*Salinarchaeum laminarum*	U976
HArcht-Ma	Laguna de Fuente de Piedra (S+B)				

*^a^(S), sediments; (B), brines.*

*^b^Culture Collection of Unique and Extremophilic Microorganisms (Moscow).*

**FIGURE 2:**
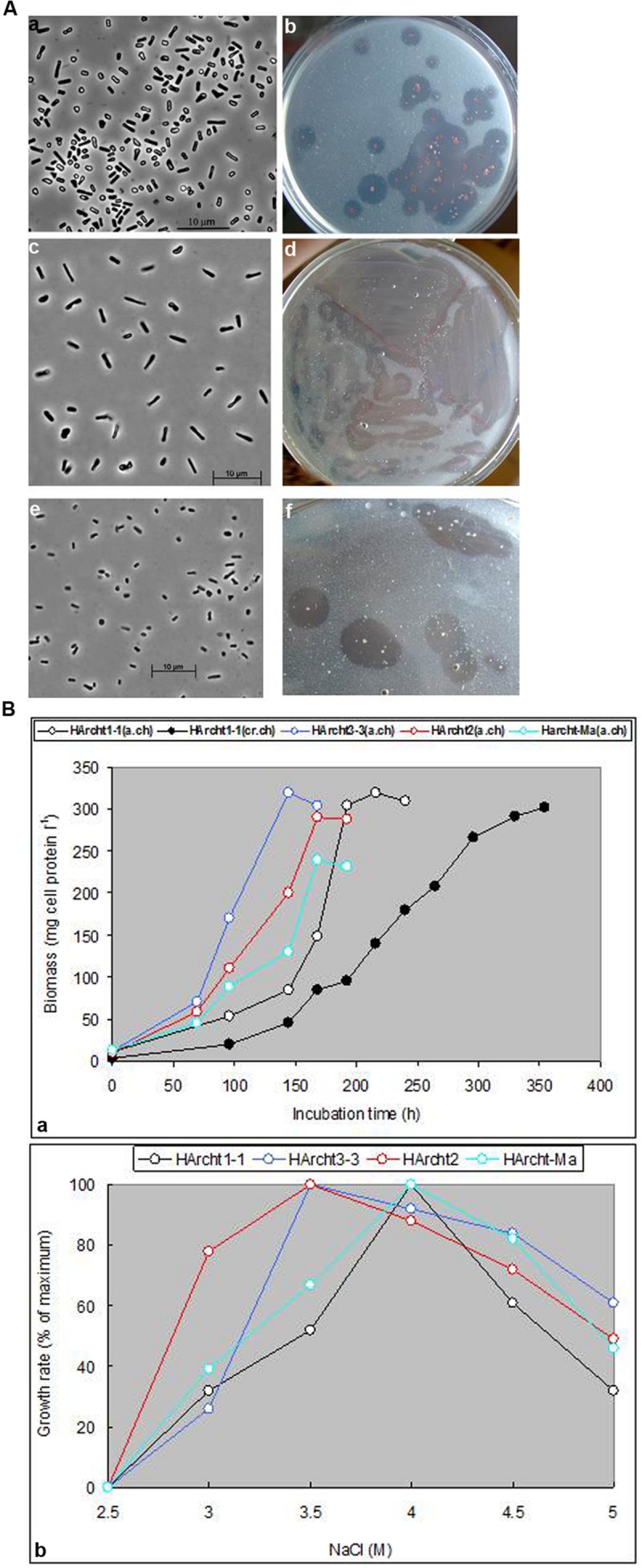
**(A)** Cell and colony morphology of haloarchaea grown on amorphous chitin at 4 M NaCl. (a,b) Strain HArcht1-2; (c,d) Strain HArcht2; (e,f) Strain HArcht-Bsk. **(B)**. Effects of NaCl on growth of haloarchaeal isolates with amorphous (a.ch) or crystalline (cr.ch) chitin as substrate.

Cells of chitin-utilizing strains were pleomorphic, varying from motile board-like (flattened) rods to non-motile coccoids. The colonies on plates with amorphous chitin were 1–2 mm, mostly dense, red or orange and with large clearance halo indicating chitin hydrolysis (**Figure 2A**). Chitin in liquid cultures at 4 M NaCl was completely degraded in 1–2.5 weeks (**Figure 2B**). The growth was initiated by cell attachment to chitin particles, followed by formation of large aggregates with subsequent gradual substrate dissolution and cell growth (Supplementary Figure S1). All strains grew with chitin at 3–5 M NaCl with an optimum at 3.5–4 M (**Figure 2B**). Microbial degradation of chitin did not lead to accumulation of reducing sugars in the supernatant. Concentration of reducing sugars in supernatants did not exceed 0.5 mM, indicating an efficient consumption of hydrolysis products (monomers and/or oligomers). Besides chitin, the only tested polysaccharide supporting growth was chitosan, which, however, was not completely depolymerized, and the growth speed was much slower. Maximal growth temperature with chitin at 4 M NaCl was 48°C for *Halomicrobium* and *Salinarchaeum* strains and 50°C for *Haloterrigena*.

Chitinase activity was measured in four isolates that represented all three genera. Amorphous chitin was used as a substrate. *Halomicrobium* and *Salinarchaeum* excreted chitinases into culture medium, while in *Haloterrigena* the activity was cell-bound. Chitinases from culture supernatant passed through 30 kDa ultrafilter, but were retained on a 10-kDa membrane (Supplementary Figure S2). *Salinarchaeum* chitinase preparation did not release measurable amounts of reducing sugars despite evident chitin degradation. The most probable explanation for this observation is that the endochitinase hydrolyzes chitin into relatively large oligomers, thus weakly increasing the concentration of reducing ends in the solution. A similar phenomenon was observed for a recently described anaerobic chitinolytic bacterium *Chitinivibrio alkaliphilus* from soda lakes (Sorokin et al., 2014). All tested chitinases were active at 1.5–5 M NaCl, with an optimal salinity 2–3 M (Supplementary Figure S2). No endoglucanase or endoxylanase activity was detected by the agar-diffusion method with CMC or birch-wood xylan as substrates in chitinase-active preparations.

### Chitinotrophic Natronoarchaea in Hypersaline Alkaline (Soda) Lakes

**FIGURE 3:**
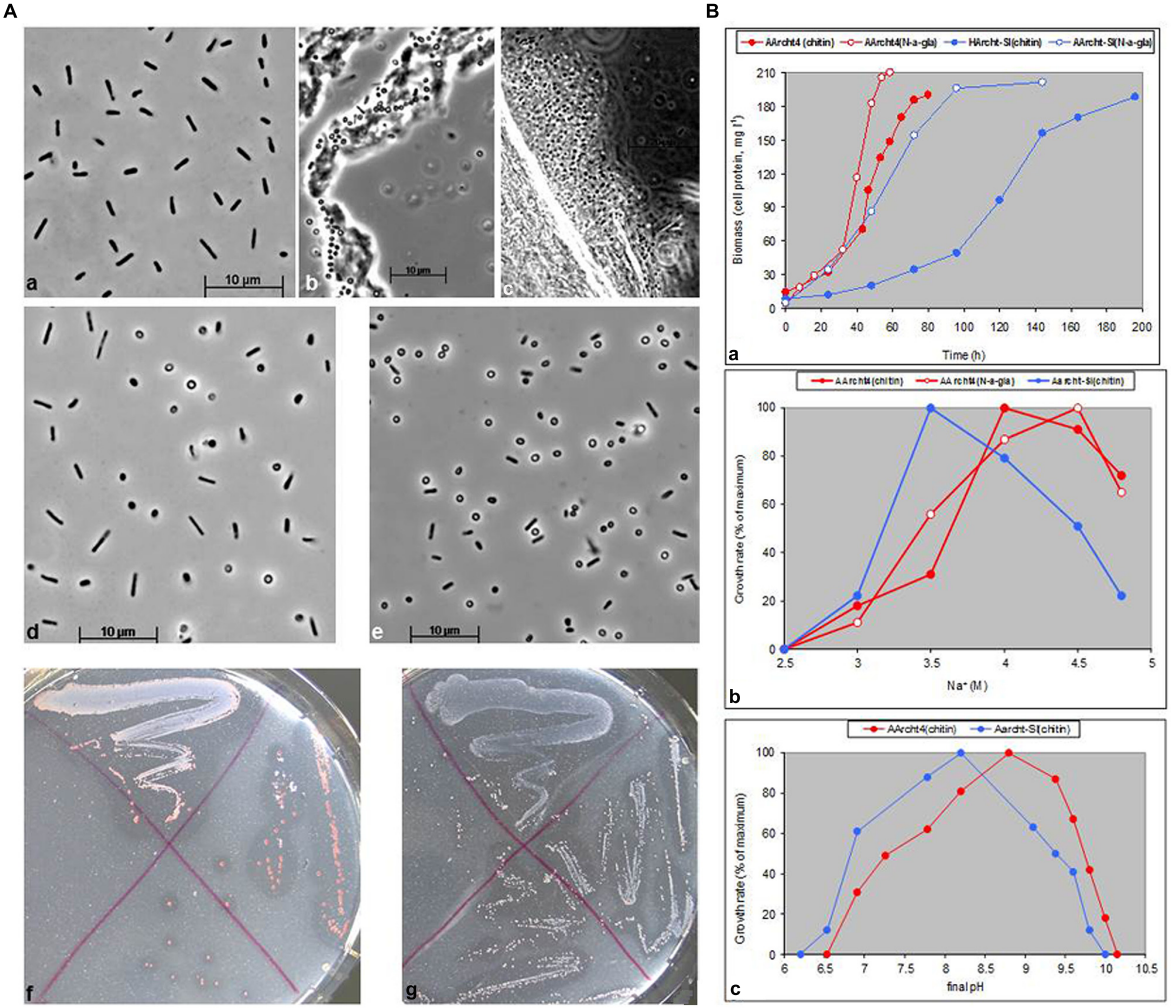
**(A)** Cell and colony morphology of natronoarchaea grown on chitin at 4 M Na^+^, pH 9.3–9.5. (a–c) AArcht4 cells in liquid culture; (a) free cells, (b,c) cells colonizing amorphous and crystalline chitin. (d,e), AArcht-Sl cells in liquid culture and in colonies. (f,g) Archt4 and AArcht-Sl colonies grown on plates with amorphous chitin. **(B)** Growth dynamics (a) and influence of sodium (b), and pH (c) on growth of natronoarchaeal isolates on amorphous chitin or *N*-acetyl-glucosamine as a sole substrates.

All twelve enrichments from brines or sediments of nine hypersaline alkaline lakes showed growth on chitin-supplemented alkaline medium C. The growth occurred within 1–4 weeks, manifested by complete substrate degradation and accumulation of pinkish biomass. Purification of initial enrichment cultures by serial dilution and replating on solid media resulted in isolation of twelve pure cultures of natronarchaeal chitinotrophs (**Table 2**). The phylogenetic analysis (**Figure 1**) indicated that they formed two new lineages of the genus level within the class *Halobacteria*: the major group included eleven and the minor group – 1 isolate, and both groups were most closely related to the genera *Halopiger* and *Natrialba*. All natronarchaeal isolates were pleomorphic motile rods in free state but tended to form coccoid cells in colonies and when aggregated on chitin particles. The cell adsorption onto chitin was clearly visible by the color change of chitin particles from white to pink (Supplementary Figure S3). The typical cell and colony morphology, and growth kinetics are shown on **Figures 3A,B**, respectively. The complete utilization of 1 g l^-1^ chitin by the natronophilic isolates was typically faster than that by neutrophilic haloarchaea. The [Na^+^] optimum for growth with chitin was at 3.5–4 M, typical for extreme halophiles. The growth pH profiling with chitin at optimal salt concentration measured for two representative strains showed that the soda lake isolate AArcht4 belongs to facultative alkaliphiles, while strain AArcht-Sl obtained from a slightly alkaline lake is an alkalitolerant halophile (**Figure 3B**). The maximal growth temperatures of strains AArcht4 and AArcht-Sl on the medium C supplemented with chitin were 41 and 43°C, respectively.

**Table 2:** Natronarchaeal chitinotrophic strains isolated from hypersaline alkaline lakes.

Strain	Source	Colony type^a^	Affiliation	Collection No. in UNIQEM
AArcht1	Soda crystallizer (brine)	1	Putative new genus 1	
AArcht3	Wadi Natrun (sediment mix)	1		
AArcht4	Wadi Natrun (brine mix)	2		U966
AArcht5	Tanatar-1 (sediment+brine	1		
AArcht6	Soda crystallizer (sediment)	2		
AArcht7^b^	Soda crystallizer (brine)	2		U967
AArcht8	Bitter-1 (sediment+brine)	1		U968
AArcht-St	Stamp Lake (sediment+brine)	1		
AArcht-Bj	Lake Badain (brine)	2		
AArcht-Mg	Lakes Hotontyn/Shar-Burdiin (sediment+brine)	1		
AArcht-Ow	Owens Lake (sediment+brine)	1		
AArcht-Sl	Searles Lake (sediment)	2	Putative new genus 2	U969

*^a^1, deep red, soft; 2, pink, solid; free cells in all isolates are thin flat motile rods; in colonies and in aggregation with chitin the cells are irregular non-motile coccoids.*

*^b^Enriched with crystalline chitin.*

Similar to neutrophilic chitinotrophic HArcht strain, natronoarchaeal isolates grew in the presence of chitosan, but not of other carbohydrate polymers tested. The chitinase activity determined by agar diffusion method or in a buffer at 4 M Na^+^ (pH 9.5) was present in culture suprnatant of strains AArcht4 and AArcht-Sl, but was very low in cell extracts (data not shown), indicating that the chitinase complex was secreted into the medium. The AArcht strains, similarly to the HArcht strains, produced very low amounts of reducing sugar equivalents when incubated with amorphous chitin, despite of obvious chitin degradation.

### Cellulotrophic Haloarchaea in Hypersaline Lakes with Neutral pH

Only three out of eight inoculates from salt lakes produced growth on insoluble celluloses and development of those positive cultures was much slower than of chitinolytic haloarchaea (1–2 months). All three cultures were obtained from mixed sediment+brine samples from lakes in Kulunda Steppe using three different forms of cellulose (**Table 3**). The original enrichments were dominated by cellobiose-utilizing haloarchaea and bacteria, with cellulotrophs present as a minor component. However, after several rounds of dilution series, it was possible to obtain pure cultures by plating maximal positive dilutions on agar medium with amorphous cellulose which allowed effective discrimination of colonies formed by cellulolytic archaea from the colonies formed by non-cellulolytic satellites. Phylogenetic analysis revealed that strain HArcel1 belongs to a new genus-level lineage closest to the genus *Halorhabdus*, whereas strains HArcel2 and HArcel3 are closely related to *Halosimplex carlsbadense*/*Halosimplex rubrum* and *Halomicrobium zhouii*, respectively (**Figure 1**).

**Table 3:** (Halo)natronarchaeal cellulotrophic strains isolated from hypersaline lakes.

Strain	Source	Cellulose type in the enrichment	Morphology		Chitin as growth substrate	Affiliation	Collection No. in UNIQEM
			Colony	Cells			
**Haloarchaea**							
HArcel1	Mixed sample, Kulunda Steppe (three salt lakes)	Amorphous	Orange, semisolid	Large irregular flat cocci	-	Putative new genus 3	U975
HArce12		Avicel	Red, semisolid	Small irregular flat cocci	-	*Halosimplex carlsbadense*	
HArcel3		Sigma 20 μm	Pink, solid	Irregular small flat cocci and rods	-	*Halomicrobium zhouii*	
**Natronoarchae**							
AArcel1	Bitter-1 (Kulunda Steppe)	Amorphous	Large, red, semisolid	Mostly large coccoids	-	*Natronolimnobius baerhuensis*	U970
AArcel2			Small, pink, solid	Mostly flat motile rods	+	Putative new genus 4	U971 U972
AArcel4	Mixed sample (Kulunda Steppe)	Avicel			+		
AArcel5		Sigma 20 μm			+		
AArcel9		Filter paper			-		
AArcel6	Lakes Hotontyn/Shar-Burdiin (Mongolia)	Amorphous			-		
AArcel7	Mixed sample (Wadi al Natrun)		Flat, pink, soft		+	Putative new genus 5	U973
AArcel8-1	Owens Lake (California)		Large, red, semisolid				
AArcel8-2			Small, pink, solid	Mostly large cocci	-	*Natronolimnobius baerhuensis*	

**FIGURE 4:**
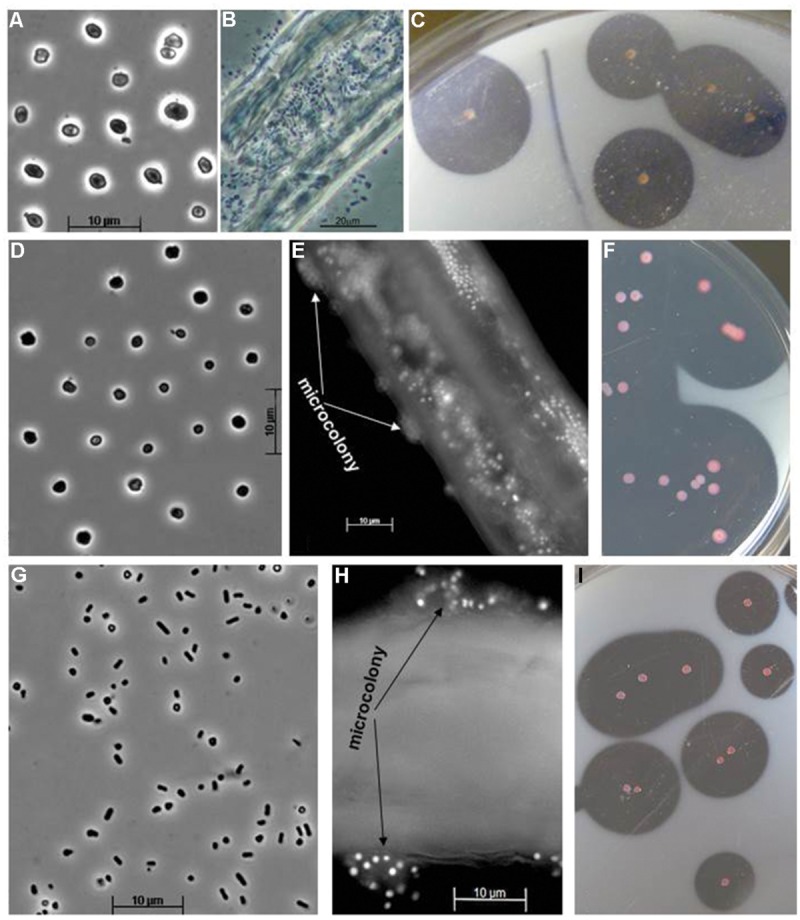
**Cell and colony morphology of cellulotrophic haoarchaea grown at 4 M Na^+^, pH 7. (A–C)**, Strain HArcel1 [**(A)** free cells, **(B)** cells colonizing cellulose fibers, and **(C)** colonies on plates with amorphous chitin]; **(D–F)** Strain HArcel2 [**(D)** free cells, **(E)** microcolony formation on a cellulose fiber, fluorescent microscopy, and **(F)** colonies]; **(G–I)** strain HArcel3 [**(G)** free cells, **(H)** microcolony formation on a cellulose fiber, fluorescent microscopy, and **(I)** colonies].

**FIGURE 5:**
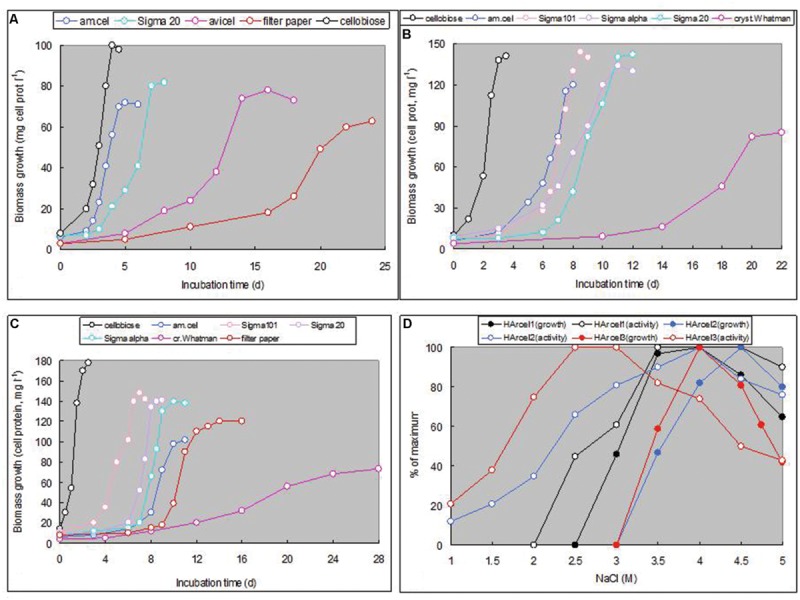
**Growth rate of three haloarchaeal strains on various forms of cellulose at 4 M NaCl, pH 7 and 37°C (**A–C**), and the effects of NaCl on growth (pH 7, 37°C, 7 days) and hydrolysis of amorphous cellulose in cell-free extracts (0.1 mg protein ml^-1^, pH 7, 37°C, 60 h) (**D**)**.

The cell and colony morphology of the haloarchaeal cellulotrophic isolates are shown on **Figure 4**. All three isolates transferred on the fresh cellulose-supplemented liquid medium first firmly attached to the surface of the cellulose particles. In the case of amorphous cellulose, this resulted in coagulation and formation of visible macro-aggregates, while in the case of crystalline cellulose the microcolony formation on the fibre and crystal surface was clearly visible by light microscopy. Growth eventually resulted in complete solubilization of cellulose particles and appearance of free cells shaped as flattened irregular cocci in most growth conditions (**Figure 4A**; Supplementary Figure S4). The tested forms of cellulose with different crystallinity were hydrolyzed by isolates with different rates, so that complete hydrolysis lasted from several days to a month. Cellobiose produced fastest growth rate, followed by growth on amorphous celluloses, while the culture progression on crystalline forms of cellulose was slowest and usually had a long lag phase (**Figures 5A–C**). An artificial, soluble CMC was not utilized as substrate. Apart from the celluloses and its dimer, all cellulolytic strains also grew on birch and beech xylanes and lichenane, and glucose. Strain HArcel2, in addition, grew on barley glucan.

Growth on amorphous cellulose occurred at a salinity range typical for extreme halophiles, 3–5 M NaCl, with the optimum at 3.5–4.5 M. The same ranges were optimal for degradation of amorphous cellulose by disrupted cells (**Figure 5D**). The cellulase activity was solely associated with cells, which was confirmed visually by an agar-diffusion method (Supplementary Figure S5). On the other hand, the endoglucanase activity, visualized with the same method with CMC as substrate, showed a very different pattern. On this substrate, endoglucanases were active in both supernatant and cell fractions and at much lower salt concentrations.

Zymography analysis has identified multiple cell-associated endoglucanases or different states of one enzyme: we observed at least 2–3 clearly separate bands in the region from 30 to 100 kDa with the most active enzymes within the range from 45 to 65 kDa (Supplementary Figure S5).

### Cellulotrophic Natronoarchaea in Hypersaline Alkaline Lakes

Positive enrichments from hypersaline alkaline lakes grown on medium C with insoluble cellulose were more diverse than from lakes with neutral pH. Overall, nine natronoarchaeal strains were isolated from five different samples (**Table 3**). Two enrichments contained two different cellulotrophic haloarchaea each, while a single dominating natronarchael organism with the ability to grow on cellulose was found in all other enrichments. Similar to the enrichments from neutral salt lakes, the microorganisms able to grow on insoluble celluloses as sole substrate represented a minor fraction from the total population of natronoarchaea, and their isolation required several rounds of plating on solid medium with amorphous cellulose as sole substrate.

**FIGURE 6:**
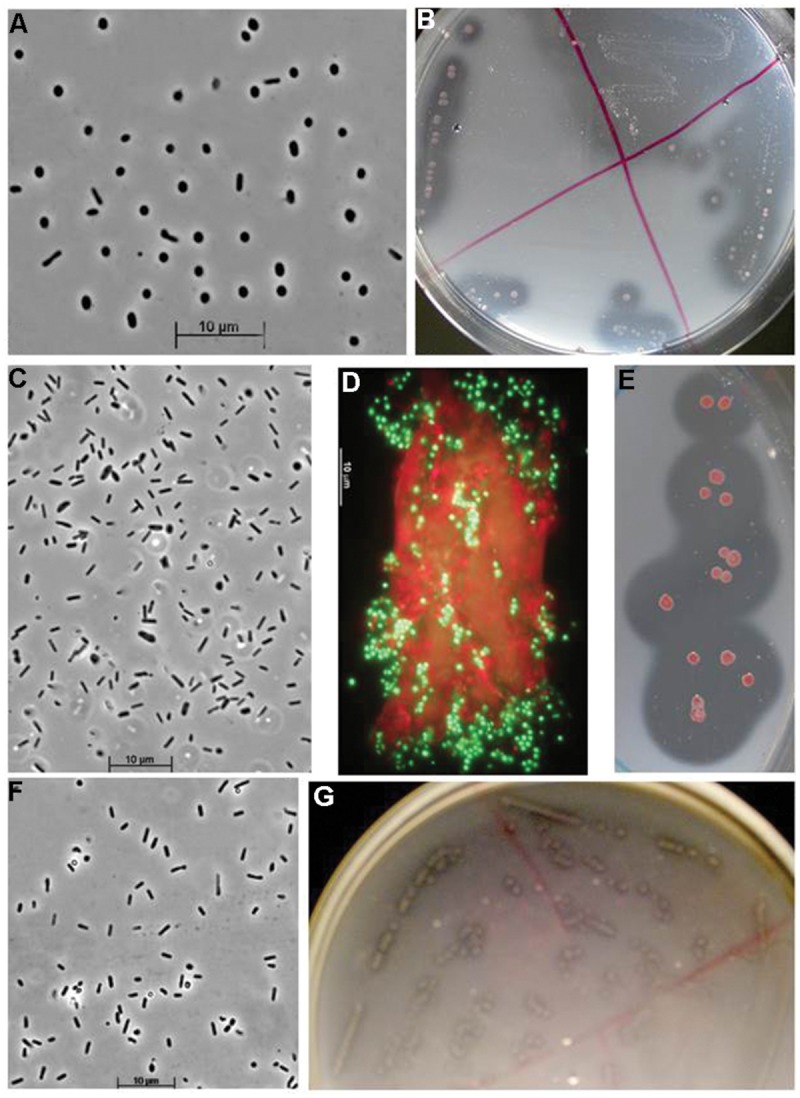
**Cell and colony morphology of cellulotrophic natronoarchaea grown on amorphous cellulose at 4 M Na^+^, pH 9.5. (A,B)** Strain AArcel1, **(C)** strain AArcel2, **(D,E)** strain AArcel9, and **(F,G)**, strain AArcel7. **(D)** Adhesion of AArcel9 cells (green) to cellulose fibrilles (red) visualized by epifluorescent microscopy using SYTO-9 staining.

**FIGURE 7:**
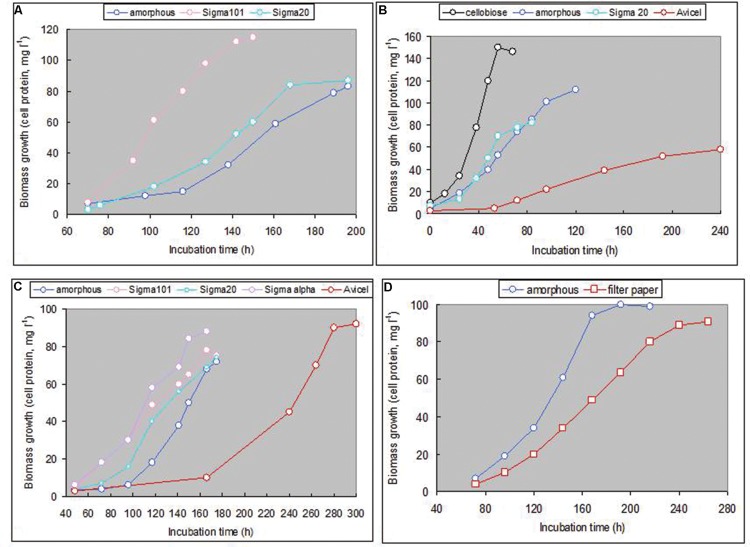
**Growth of natronarchael strains on various forms of cellulose at 4 M Na^+^, pH 9.5 and 37°C. (A)** strain AArcel1, **(B)** strain AArcel2, **(C)** strain AArcel7, and **(D)** strain AArcel9.

The obtained isolates could be divided into two groups based on their colony and cell morphology. Seven isolates of the first group formed tiny pink colonies that had a large clearance zone of cellulose degradation; the cells grew in liquid cultures mostly as thin flat motile rods. The second, minor group, represented by only two strains, formed large red-orange colonies with relatively small clearance zone. The cells of the second group were large cocci in both, liquid culture and colonies (**Figure 6**). Phylogenetic analysis has revealed that the dominant group represents a new genus-level lineage related to the genus *Halovivax*, while the first representative of the minor group is closely related to *Natronolimnobius baurhaensis* and the second one to *Halopiger* representatives and the majority of AArcht strains (**Figure 1**). These two groups differ in their hydrolytic potential. The major group grew on all forms of cellulose tested (although the growth on crystalline forms was very slow). The minor group utilized only amorphous cellulose (**Figure 7**). All isolates grew well using birch and beech wood xylanes or cellobiose. Interestingly, the closely related to one of the strains of the minor group the type strain of *Natronolimnobius baerhaense* grew well on xylanes and Sigma celluloses 101 and 20 μ, but not on amorphous or crystalline forms. Similarly to HArcel isolates, AArcel strains did not grow on CMC and growth on native celluloses started with firm adhesion of cells to cellulose particles (**Figure 6**).

**FIGURE 8:**
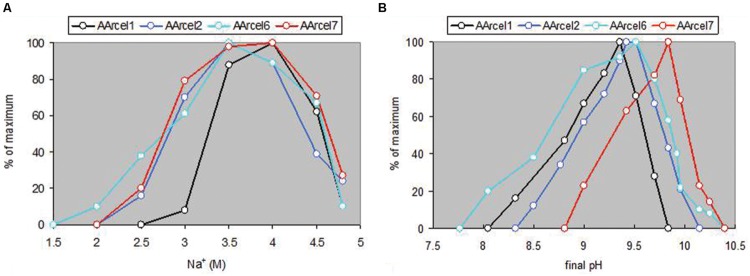
**Effects of salinity **(A)** and pH **(B)** on growth of natronoarchaeal isolates cultured on amorphous cellulose for 7 days at 37°C. (A)** pH 9.5 and **(B)** salinity is 4 M of total Na^+^.

AArcel strains grew on amorphous cellulose at conditions typical for extreme haloalkaliphiles, total Na^+^ concentration ranging from 2.5 to 4.5 M with the optimum at ∼4 M. All strains, except AArcel7, were obligate moderate alkaliphiles, growing at pH 8.0–9.8 with optimal pH at ∼9.5; (AArcel7 grew optimally at pH>10) (**Figure 8**; Supplementary Figure S6). The optimal growth temperature on all substrates was 36–38°C with a maximal tolerated temperature of 42–43°C. Remarkably, four out of seven isolated natronoarchaeal cellulotrophs (**Table 3**) were able to use chitin as growth substrate, apart from cellulose and xylane, which is a rare combination. Moreover, five of seven AArcel isolates were able to grow on starch and/or amylopectin (branched α-1,4, α-1,6 glycans).

Analogously to a previously discussed observation for HArcel strains, measurements of cellulase activity in cell fractions of the AArcel strains by monitoring reducing sugar release did not detect a significant accumulation of sugars. The endoglucanase activity of AArcel strains, however, was clearly detectable by the agar-diffusion method. Detected activity was predominantly cell-associated (Supplementary Figure S7). Another similarity to HArcel strains is that the cell-associated cellulases were active at higher salt concentrations than that present in the culture broth. When the cells were pre-grown with amorphous cellulose, the level of endoxylanase activity was very low, suggesting that the endoglucanase activity was cellulose-specific. The endoglucanases from AArcel strains were profoundly alkalitolerant. Zymography conducted under mildly denaturing conditions produced positive results only for a cell-associated fraction of one (AArcel4) out of three tested strains. Three bands were observed in the region from 35 to 60 kDa, with the most active enzyme being ∼60 kDa (Supplementary Figure S8). Analogous analysis using PAGE at non-denaturing conditions demonstrated a presence of endoglucanases in two other strains, AArcel1 and AArcel7. This indicates that AArcel1 and AArcel7 endoglucanases denature at milder conditions than AArcel4 endoglucanases (data not shown).

## Discussion

Our data demonstrate for the first time that extremely halophilic euryarchaea can be enriched and isolated from their natural hypersaline habitats using native forms of insoluble chitin and cellulose as specific growth substrate at salt-saturating conditions at both neutral and alkaline pH. Although the ability of neutrophilic haloarchaea to hydrolyze cellulose and chitin was reported previously, this type of metabolism has not been demonstrated for natronoarchaea.

The chitinotrophic halo(natrono)archaea seems to belong to very narrow specialists with chitin, chitosane and their monomers as the only utilizable growth substrate, while all cellulotrophic halo(natrono)archaea apart from the different forms of insoluble celluloses also utilize xylanes. Furthermore, the finding that some of the cellulotrophic natronoarchaeal isolates can grow both on celluloses and chitin seems to be outstanding. Such an ability is rarely combined in a single organism and, to our knowledge, has not been reported previously in the whole Archaea kingdom. It should be noted though that several publically available genomes of haloarchaea encode both putative chitinases and cellulases (Supplementary Table S1), suggesting that these archaea might belong to the group of cellulo/chitino-trophic halo/natronoarchaea identified in this work.

One of the interesting finding in regard to the cellulotrophic isolates is that use of different forms of insoluble cellulose in the enrichment resulted in selection of different dominant organisms that, apparently, were better adapted to the specific structural property of the polymers with otherwise identical chemical nature. The discovered inability of these isolates to use CMC as a substrate strongly suggests that this artificial soluble form of cellulose should not be used as a growth substrate in environmental studies. This finding suggests that some conclusions based on experiments that use CMC might require revision. This concern was also expressed in a recent review by Koeck et al. (2014). Our results on measuring the localization of cellulase activity also showed a clear difference between CMCase and degradation of insoluble cellulose. The endoglucanases hydrolyzing CMC were active in both supernatant and cell fractions and at low salt concentrations, while only the cell-bound cellulases hydrolyzed insoluble cellulose. This might be an indication of complex mechanism of cellulose degradation, involving various GHs with different localization and parameters of catalytic activities.

Another unusual feature of the studied halo(natrono)archaea is that growth on chitin and, especially, on celluloses was not accompanied by a significant release of reducing sugars into the medium, which is often considered as an indication of cellulolytic activity. This might be explained by a fundamental difference between the cellulotrophic and cellulolytic strategies: while the former has evolved to use the polymer for growth without losing the hydrolysis products to competitors, the latter is not necessarily growth-dependent. These opportunistic microorganisms have enzymes for complete hydrolysis of soluble oligosaccharides, formed by the previous group from insoluble substrate.

Another obvious conclusion is that chitinotrophic halo(natrono)archaea are easier to enrich from hypersaline habitats than cellulotrophic halo(natrono)archaea. This likely reflects the higher abundance of chitin produced by brine shrimps *Artemia* while plant cellulose is a scarce carbohydrate polymer in these environments, as it has to come from surrounding land areas.

Future research will study genomics of four discovered groups of polysaccharide-utilizing haloarchaea and biochemical properties of their hydrolytic enzymes.

## Conflict of Interest Statement

The authors declare that the research was conducted in the absence of any commercial or financial relationships that could be construed as a potential conflict of interest.
