# Supplementary material for: Halo(natrono)archaea isolated from hypersaline lakes utilize cellulose and chitin as growth substrates
Source: Front Microbiol. 2015 Sep 15;6:942. doi: 10.3389/fmicb.2015.00942 (PMC4569967; doi:10.3389/fmicb.2015.00942)
Supplement: Supplementary file 1 [file Image_1.PDF]

Supplementary table 1. Distribution of genes, encoding GHs, potentially involved in hydrolysis of chitin, cellulose and its derivatives among all haloarchaeal genomes, present in the CAZy database.

Species	Chitinases	Cellulases and related glucanases					
	GH 18	GH3	GH5	GH9	GH12	GH16	GH94
Halalkalicoccus jeotgali B3	-	2	-	-	-	-	-
Haloarcula hispanica ATCC 33960	-	3	-	-	-	-	-
Haloarcula hispanica N601	-	3	-	-	-	-	-
Haloarcula marismortui ATCC 43049	-	2	-	-	-	-	-
Haloarcula sp. CBA1115	-	3	-	-	-	-	-
Haloferax volcanii DS2	-	2	-	-	-	-	-
Halopiger xanaduensis SH-6	-	8	-	-	-	-	-
Halorhabdus tiamatea SARL4B	-	6	6	1	1	-	3
Halorhabdus utahensis DSM 12940	-	7	8	1	-	-	2
Halostagnicola larsenii XH-48	-	3	1	-	-	-	-
Halovivax ruber XH-70	-	2	-	-	-	-	-
Natronococcus occultus SP4	-	2	-	-	-	1	-
Halobacterium salinarum R1 DSM 671	2	-	-	-	-	-	-
Halobacterium sp. NRC-1	2	-	-	-	-	-	-
Haloferax mediterranei ATCC 33500	4	1	-	-	-	-	-
Halogeometricum borinquense DSM 11551	4	1	-	-	-	-	-
Halomicrobium mukohataei DSM 12286	7	1	-	1	-	-	-
Haloterrigena turkmenica DSM 5511	1	2	3	-	-	-	-
Natrinema sp. J7-2	4	1	-	-	-	-	-
Salinarchaeum sp. Harcht-Bsk1	5	3	4	1	-	1	-

No genes, coding for GH6, 7, 8, 19 and 30 were found in archaeal genomes, according to CAZy.

Supplementary table 2. Hypersaline lakes used as inoculum and their key chemical characteristics

Lake	Location		Total salts (g/l)	pH	Soluble carbonate alkalinity (M)
	Area	Coordinates			
**Hypersaline salt lakes**					
Cock Salt Lake	Kulunda Steppe (Altai, Russia)	N52^{o}16'/ E79^{o}52'	280-320	7.5-7.7	-
Lake Lomovoe		N51^{o}42'/ E79^{o}42'	300-340	7.9-8.1	-
Hummocky Lake		N51^{o}42'/ E79^{o}56'	300-350	8.0-8.1	-
Lake Elton	South Russia	N49^{o}10'/ E46^{o}39'	320	6.7	-
Lake Baskunchak		N48^{o}14'/ E46^{o}35'	360	6.2	-
Crimean salt lakes	Crimea (Russia)	N45^{o}02'/ E36^{o}11'	150-200	7.2-8.1	-
Barun Davst Nur	n-e Mongolia	N48^{o}28'/ E113^{o}12'	210	8.1	-
Laguna de Fuente de Piedra	Malaga (Spain)	N37º06'/W04º46'	205	8.0	-
**Hypersaline alkaline and soda lakes**					
Tanatar-1	Kulunda Steppe (Altai, Russia)	N51^{o}39'/ E79^{o}48'	400	11.0	5.0
Bitter-1		N51^{o}40'/ E79^{o}54'	330	10.3	4.0
Trona crystallizer		N51^{o}39'/ E79^{o}46'	380	9.6	3.1
Stamp Lake		N51^{o}41'/ E79^{o}46'	240	9.1	0.15
Shar-Burdiin, Hotontyn	n-e Mongolia	N48^{o}50'/ E113^{o}51'	220-360	9.6-9.9	0.9-1.2
Lake Badain (lagoon)	Badain-Jaran desert (Inner Mongolia)	N39º33/E102º21'	495	9.7	1.4
Hamra, Fazda, Beidah, Gaar, Umm-Risha, Zugm, Ruzita, Khadra	Wadi al Natrun (Libyan Desert, Egypt)	N30º24'/E30º18'	200-360	9.1-9.9	0.1-0.9
Searles Lake	California (USA)	N35º44'/W117º20'	350	9.8	0.2
Owens Lake	California (USA)	N36º26'/W117º57'	180	9.7	1.0

Growth of haloarchaea on chitin in liquid cultures

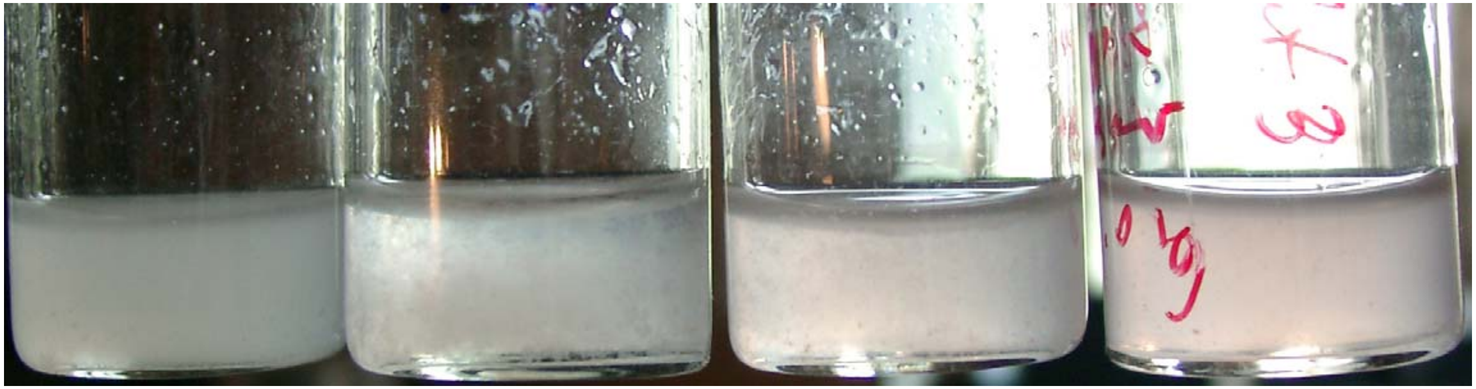

Progressive growth on amorphous chitin of strain HArcht 3-3

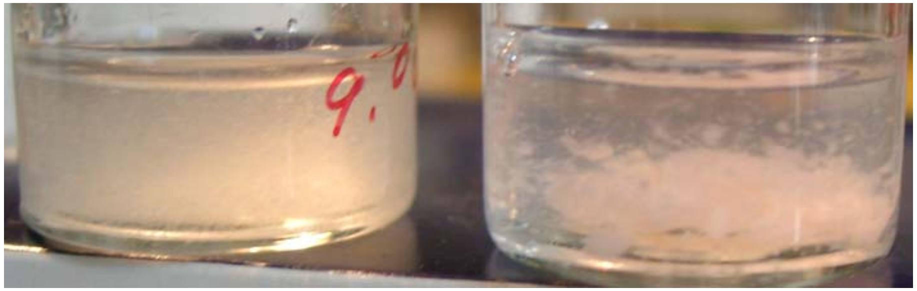

Growth on crystallin chitin (HArcht3-3)

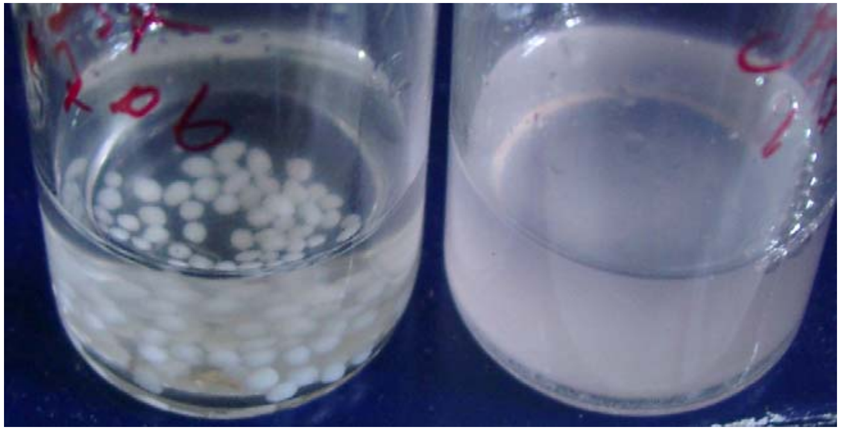

HArcht 1-2 HArcht 2

Growth on amorphous chitin of two different types of attacking

Supplementary Fig.S1: Haloarchaeal attack of insoluble chitin in liquid cultures

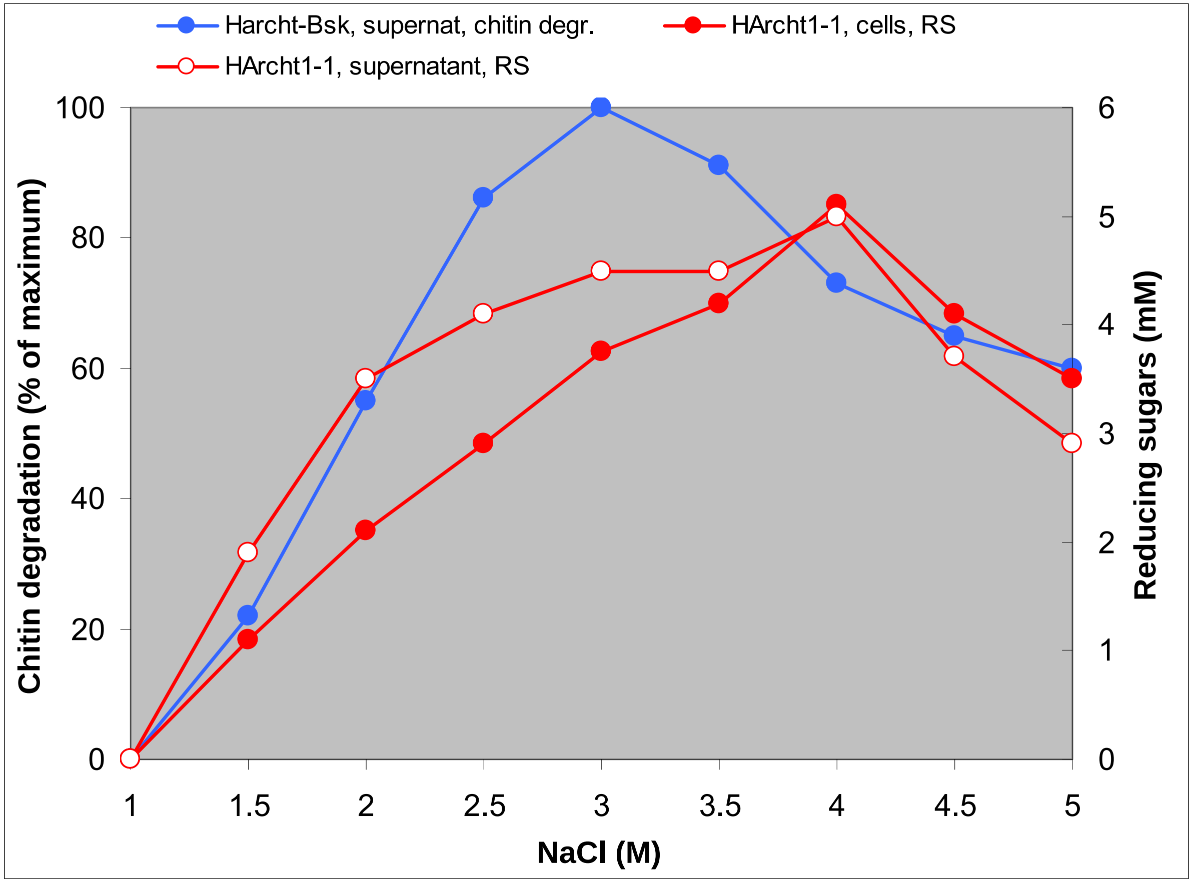

Supplementary Fig.S2A: Influence of NaCl on chitinase activity at pH 7 and 37 °C in cell fractions of HArcht strains accessed either by release of reducing sugars from amorphous chitin or by the degree of its depolymerization (optical density). The cell-free extracts were obtained by sonication, the supernatants were first filtered through 0.2 mm filter and then concentrated x20 times by ultrafiltration on 10kDa membrane. The final protein concentrations in cell extracts were 0.1 mg/ml and in supernatatns – 0.02 mg/ml, incubation time – 72-96 h.

Halomicrobium HArcht 1-1

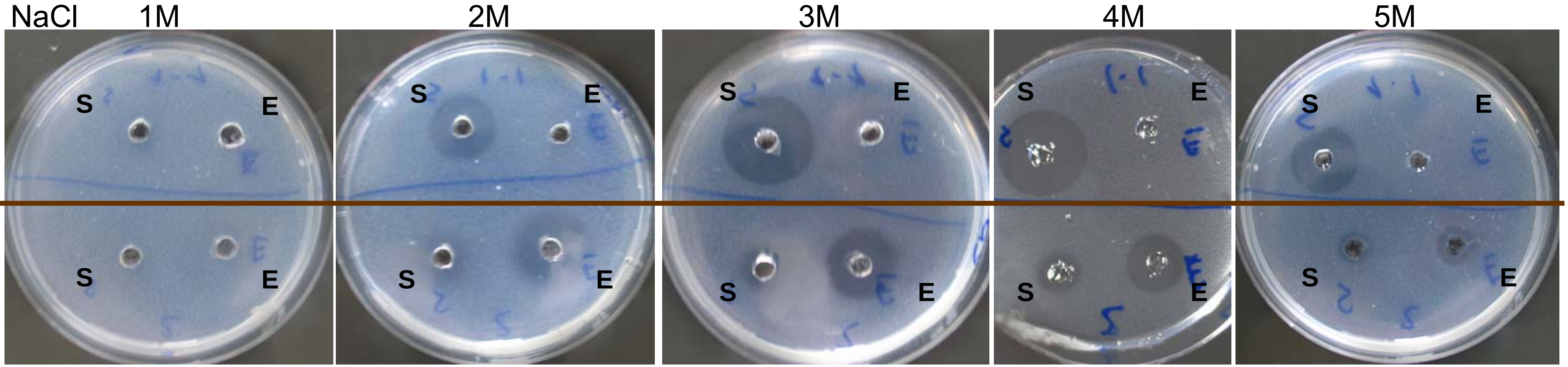

Haloterrigena HArcht 2

Supplementary Fig.S2B. Influence of NaCl on chitinase activity in cell fractions of strains *Halomicrobium* HArcht 1-1 (top half) and *Haloterrigena* HArcht2 (bottom half) tested by agar-diffusion method. The cells were grown with amorphous chitin at 4 M NaCl.**E** –cell-free extract (50 µg prot); **S** – culture supernatant concentrated over 10kDa membrane for x20 times (4 µg prot). Incubation time=60h, t=30oC.

Conclusions:

1 – In HArcht1-1 the activity is located **in supernatant** and is working at 2-5 M NaCl with an **optimum at 3-4 M**

2 – In HArcht2 the activity is **cell-associated** and working at 2-4 M NaCl with an optimum at **2-3 M**

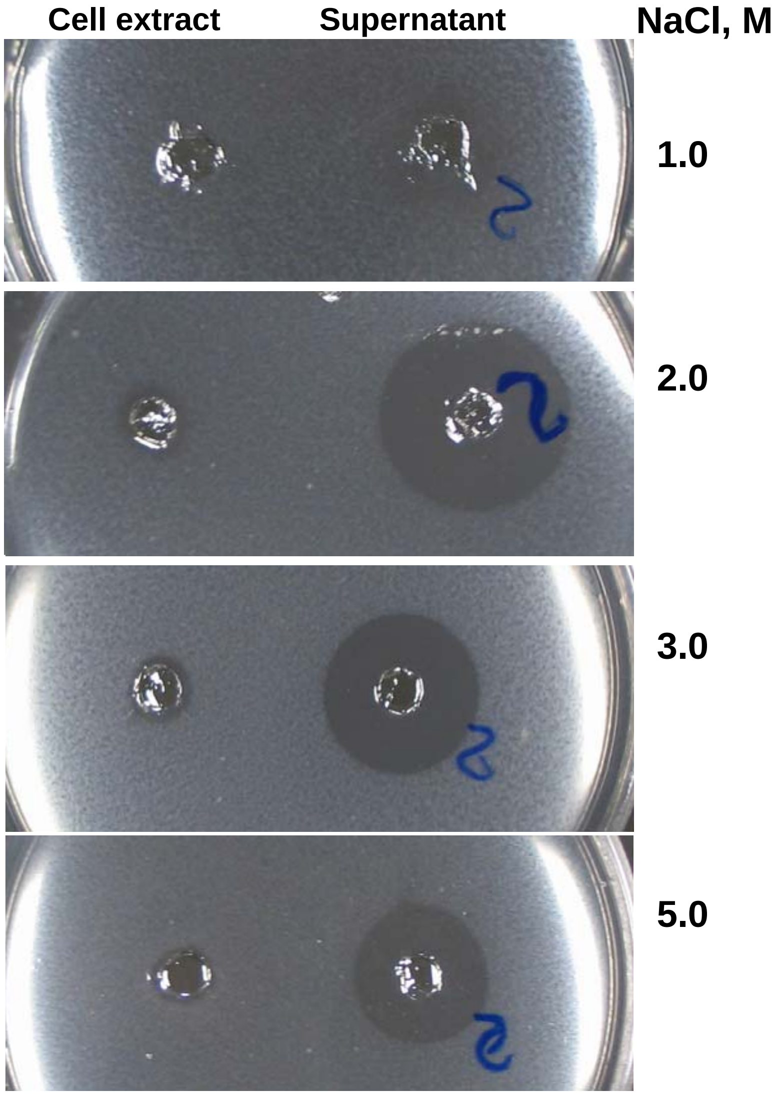

Supplementary Fig.S2C. Influence of NaCl on chitinase activity in cell fractions of strains *Halomicrobium* HArcht 3-3 tested by agar-diffusion method. The cells were grown with amorphous chitin at 4 M NaCl. Cell-free extract (50 µg prot); supernatant concentrated over 10kDa membrane for x20 times (4 µg prot). Incubation time=60h, t=30°C.
Conclusions: the activity is located **in the supernatant** and is active at 2-5 M NaCl with an **optimum at 2 M**

NaCl (M)

1.0 1.5 2.0 2.5 3.0

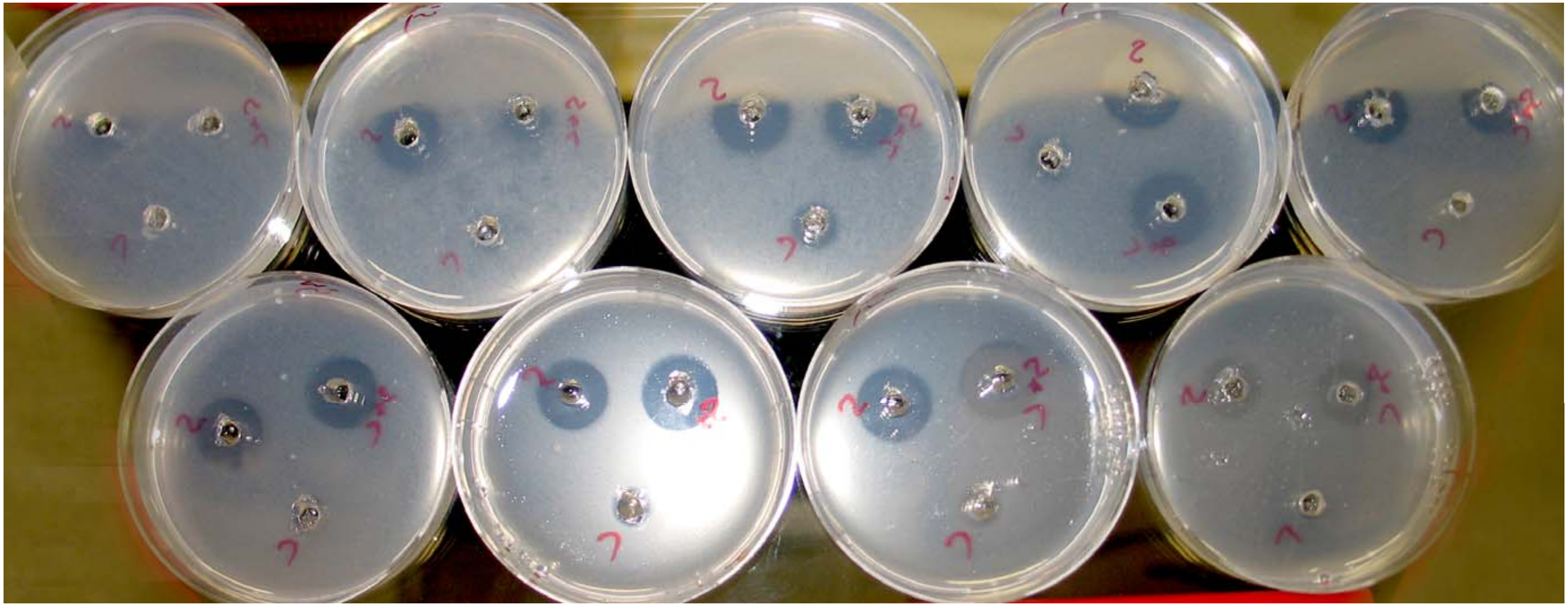

3.5 4.0 4.5 5.0

Supplementary Fig.2D: Influence of NaCl at pH 7 on chitinolytic activity in culture fractions of *Salinarchaeum* **HArcht-Bsk.** Incubation time: 60h at 30°C. Left spot: culture supernatant x20 times concentrated on 10 kDa ultramemberane (protein 4 μkg); right spot: culture supernatant+cell extract(50 μkg protein); Bottom spot: cell extract.

Conclusion: The activity is located in the **supernatant** and the chitinase is active within the NaCl range from 1.5 to 5 M (opt. at 2-2.5 M).

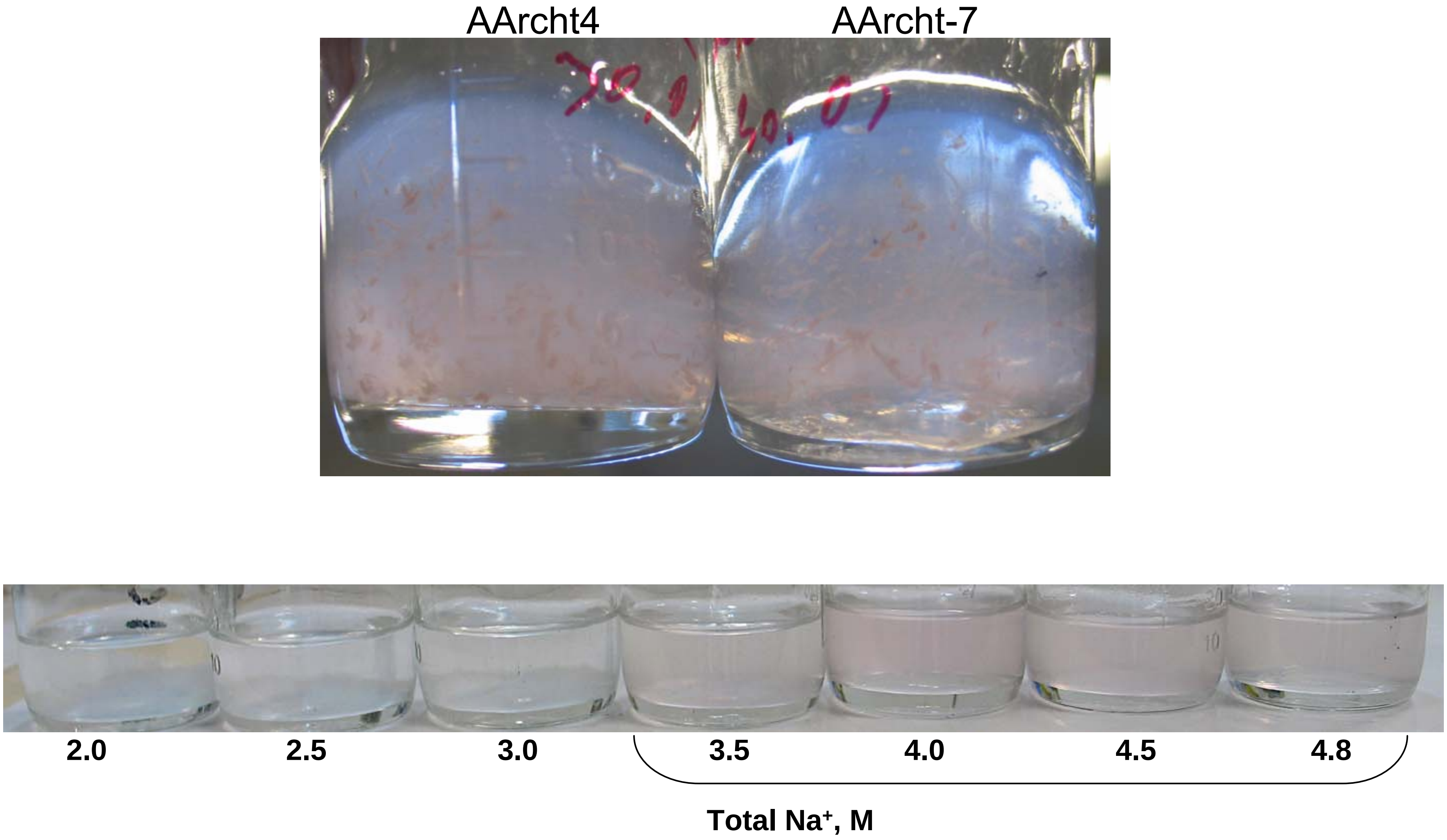

Supplementary Fig.S3. Top: Colonization of cyistallin chitin particles by natronoarchae at 4 M Na^+, pH 9.5. Bottom: Influence of Na+ at pH 9.5 on growth of strain AArcht4 on amorphous chitin at 37°C (3 days).

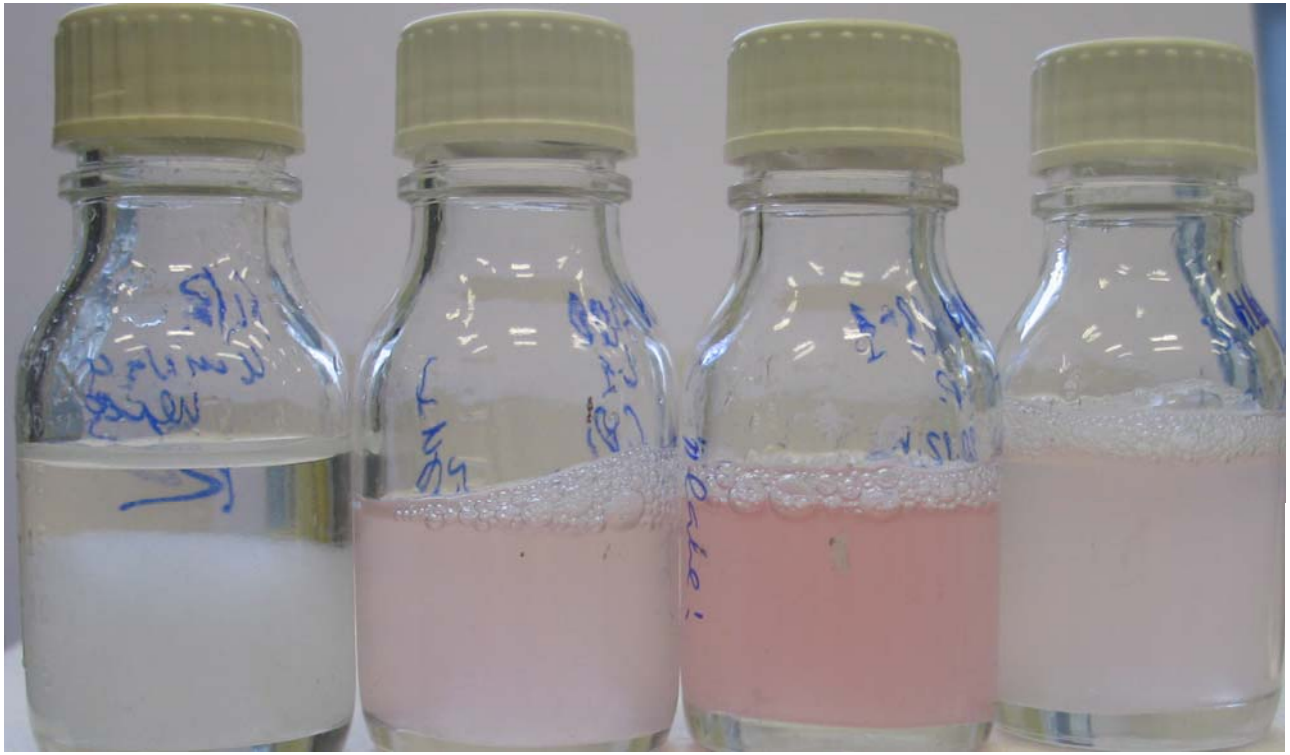

Control **HArcel2** **HArcel3** **HArcel1**

NaCl, M 3.0 3.5 4.0 4.5 5.0

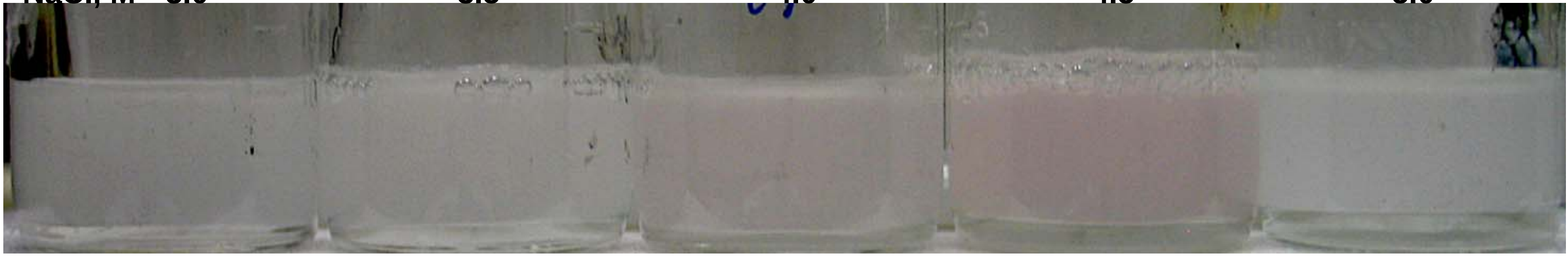

Supplementary Fig.S4 Top: Growth of haloarchaea on Whatman filter paper cellulose fibres at 4 M NaC, pH 7 (3-4 weeks); Bottom: influence of NaCl at pH 7 on growth of strain **HArcel2** with amorphous cellulose (incubation time: 7 days)

HArcel1: incubation 10 days

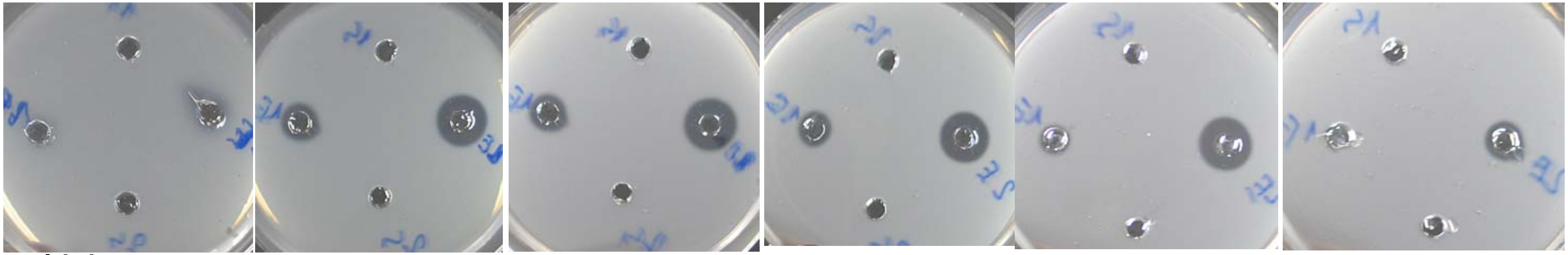

NaCl (M): 1.0 2.0 3.0 4.0 4.5 5.0

HArcel2 (left) and **HArcel3** (right): incubation 18 days

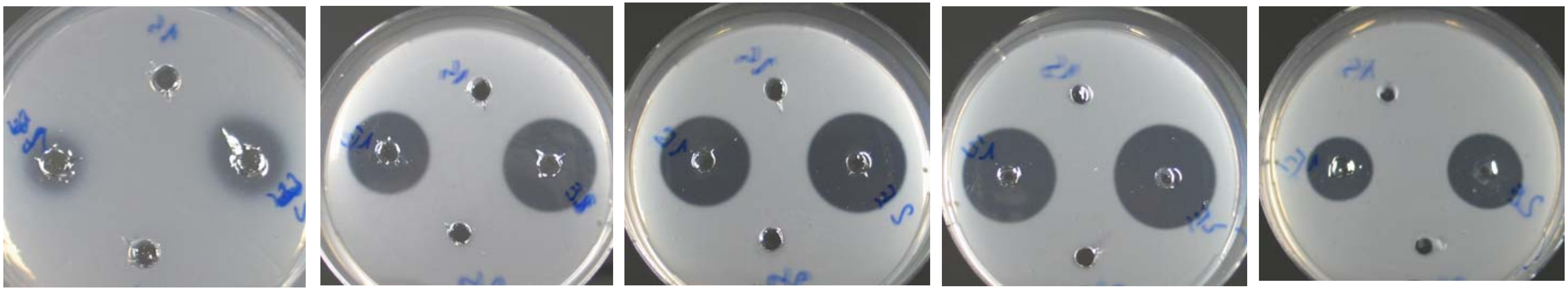

NaCl (M): 1.0 2.0 3.0 4.0 5.0

Supplementary Fig.5SA: Cellulase activity (hydrolysis of amorhous cellulose) in fractions of HArcel strains **grown on am.cellulose**. Middle – cell free extracts (100-120 µg protein). Top and bottom: supernatants concentrated on 10kDa filter.

NaCl 1M 2 M 3 M 4M 5M **KCl** 4 M

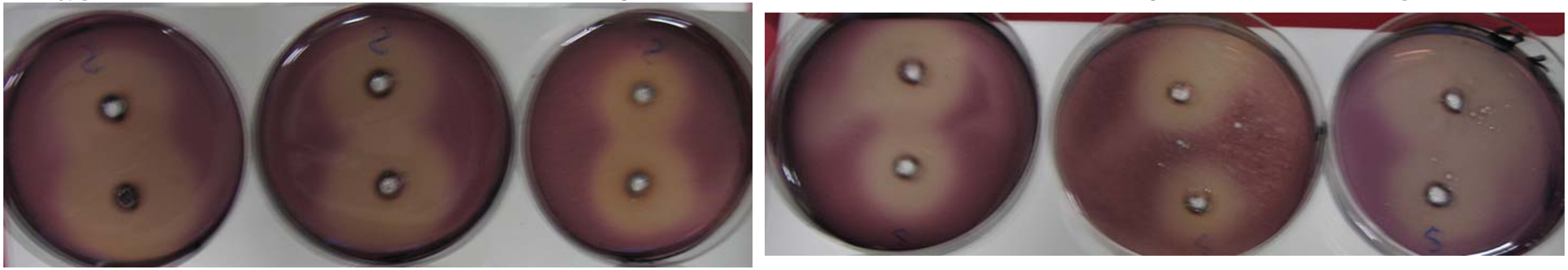

Endocellulase activity strain **HArcel2** grown on amorphous cellulose at 4 M NaCl. The plates were incubated for 65 h at 37ºC. Supernatant concentrate, 10kDa (top raws; 20 µg protein); cell-free extract (bottom raw), 90 µg prptein).

NaCl (M) =1.0 2.0 3.0 4.0 5.0

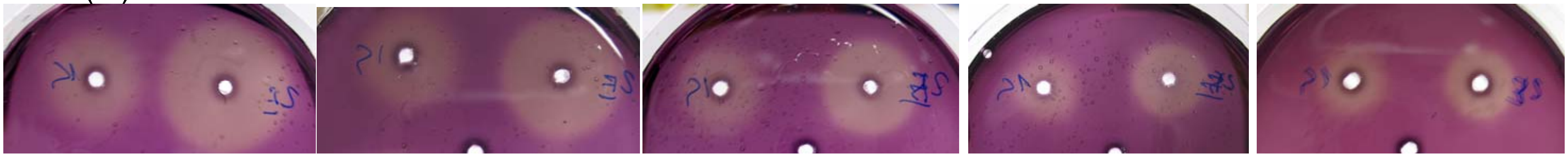

Endocellulase activity in **HArcel 3** strains grown on am.cellulose at 4 M NaCl. Incubation: 60 h at 35ºC. Cell extract (left; 100 µg prot); right - supernatant concentrated times on 10kDa filter (15 µg protein). The activity is present both in cells and in solution and it is relatively salt-sensitive.

Supplementary Fig. S5B: Endocellulase activity in fractions of HArcel strains visualized by agar-diffusion method

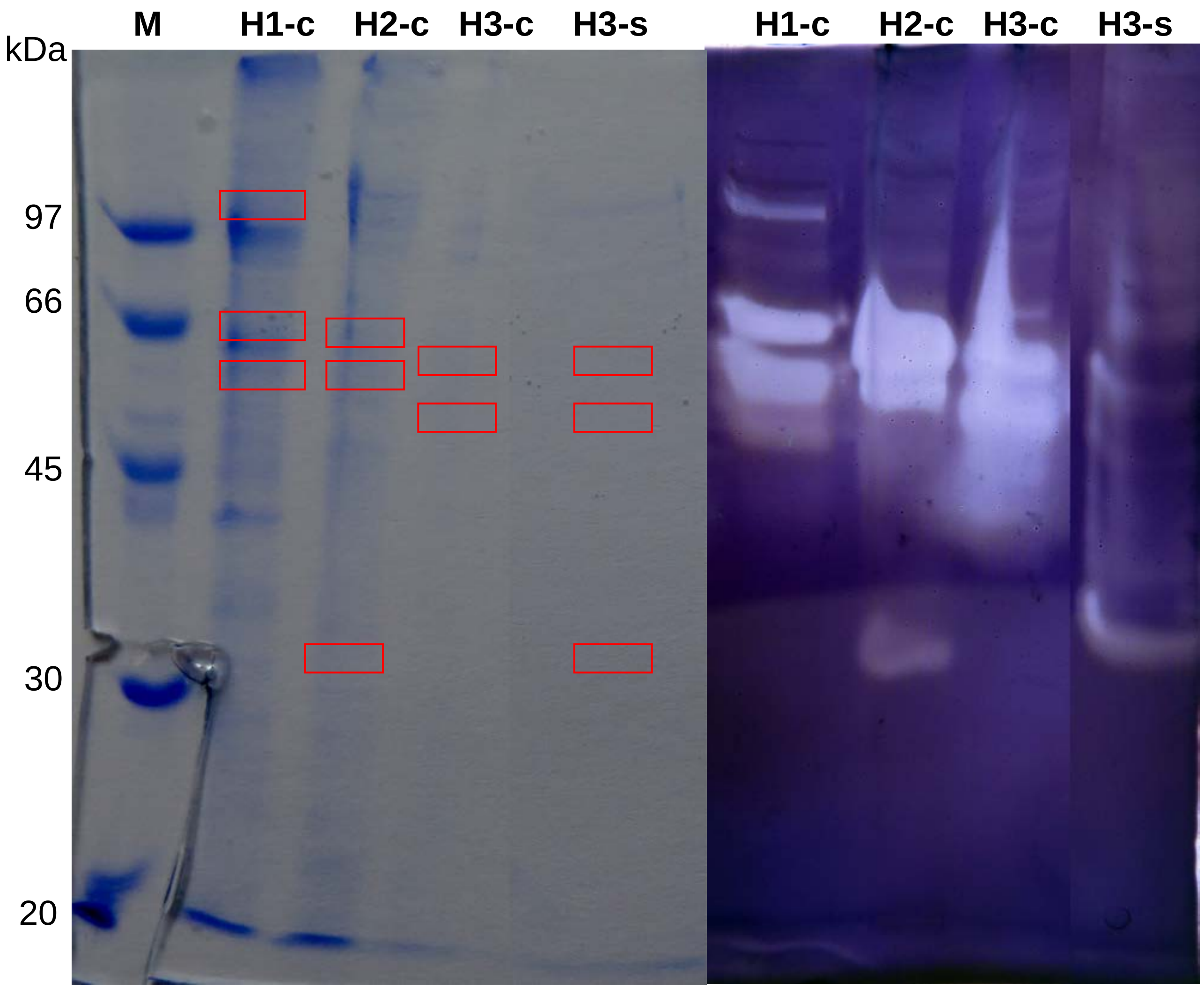

Supplementary Fig.S5C

Endocellulases in denaturing PAGE (10%, 0.1% CMC). Proteins were denatured at 46ºC for 2 h in Tris-HCl, pH 8 with 3% SDS. Renaturation at 4ºC by two 30 min wash in Tris-HCl, pH 8 with 25% 2-PrOH followed by two washes without 2-PrOH. The activity was developed in 12 h at 37ºC in 2 M NaCl, pH 7.

H1-c=**HArcel1**, cell extract; H2-c=**HArcel2**, cell extract; H3-c=**HArcel3**, cell extract; H3-s=**HArcel3**, supernatant, x20 concentrated on 10kDa membrane.

AArcel1: growth with amorphous cellulose at pH 9.5 and 37°C (7 days): salt profile

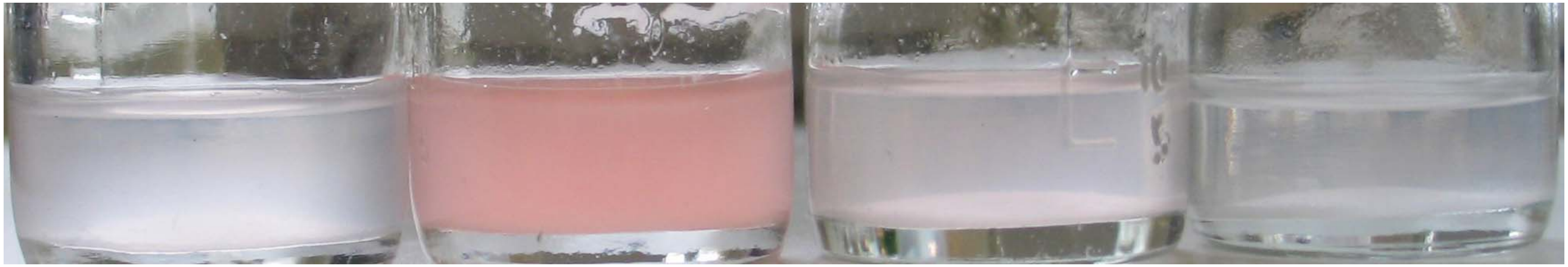

Total Na^{+}(M): **3.0** **4.0** **4.5** **4.8**

AArcel2: growth with amorphous cellulose at pH 9.5 and 37°C (5 days): salt profile

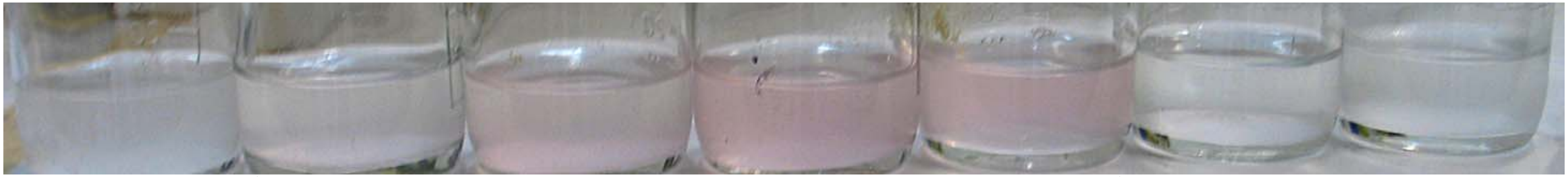

2.0 **2.5** **3.0** **3.5** **4.0** **4.5** **4.8**

Total Na^{+}, M

AArcel2: growth with amorphous cellulose at 4 M total Na^{+}, and 37°C (5 days): pH profile

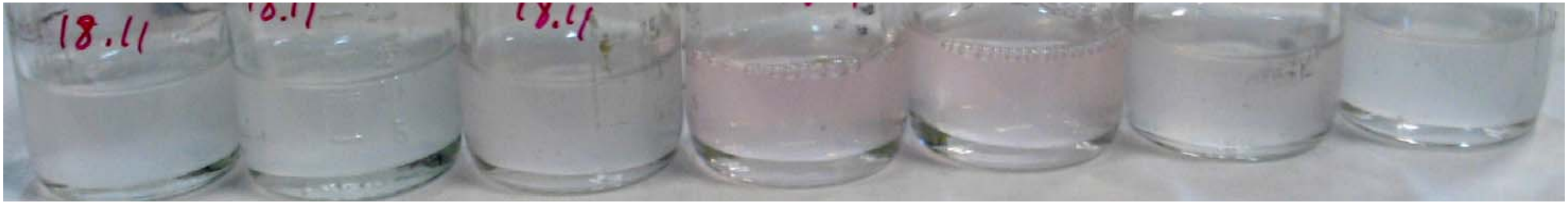

8.77 **9.0** **9.47** **9.62** **9.73** **9.91** **10.30**

Final pH

Supplementary Fig.S6: Examples of growth of AArcel strains with amorphous cellulose at different salt and pH.

β 1,4-endoglucanase (CMC)

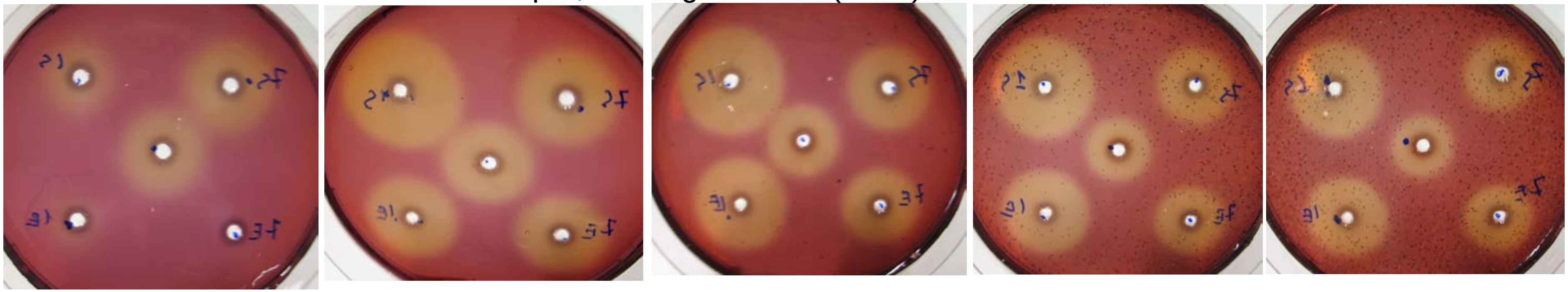

β 1,4-endoxylanase (birch-wood xylane)

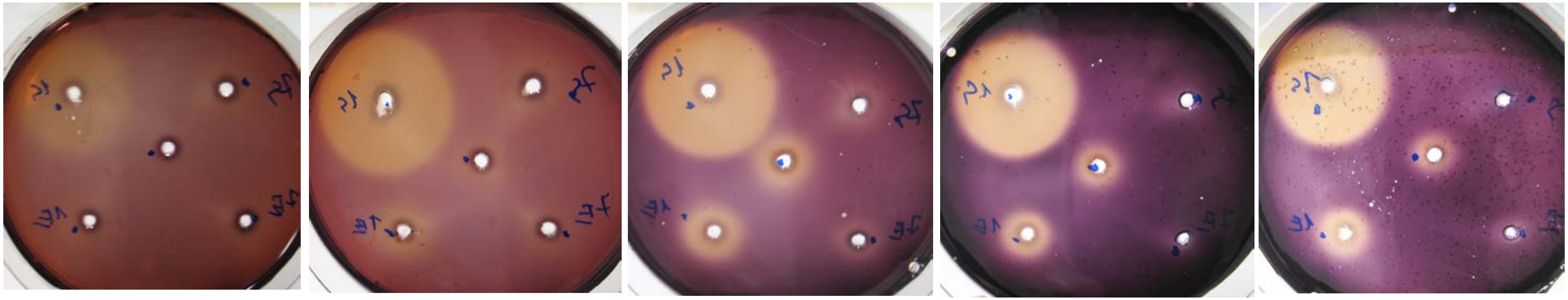

Na^+ (M) **1.0** **2.0** **3.0** **4.0** **4.5**

Influence of salt on endocellulase and endoxylanase activities in cell fractions of natronoarchaea grown with amorphous cellulose at 4 M Na^+, pH 9.5. Assay conditions: pH 9.5,incubation 48h at 37°C. Top: **supernatant**, concentrated on 10kDa(15 µg prot); bottom and centre – s**onicated cells** (40 µg prot). Left: strain **AArcel1**; centre – strain **AArcel4** right: strain **AArcel7**;

pH **7.5** **8.0** **8.5** **9.0** **9.5** **10**

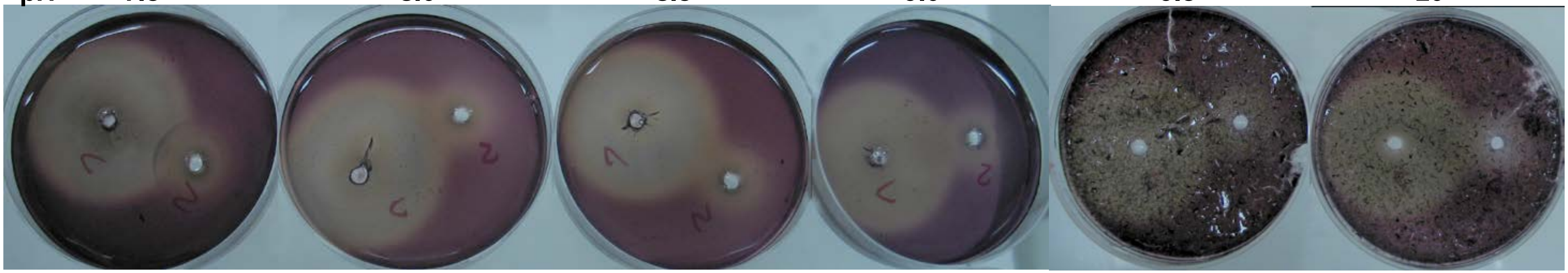

Influence of pH at 4 M Na^+ on endocellulase activity in cell fractions of **HArcel4** grown with amorphous cellulose at pH 9.5. Assay conditions: incubation at 4 M Na^+, 72h at 37°C. : left, s**onicated cells** (40 µg prot); right, **supernatant**, concentrated on 10kDa(20 µg prot).

Supplementary Fig.7S: Influence of Na+ and pH on hydrolytic activity in cell fraction of cellulotrophic natronoarchaea

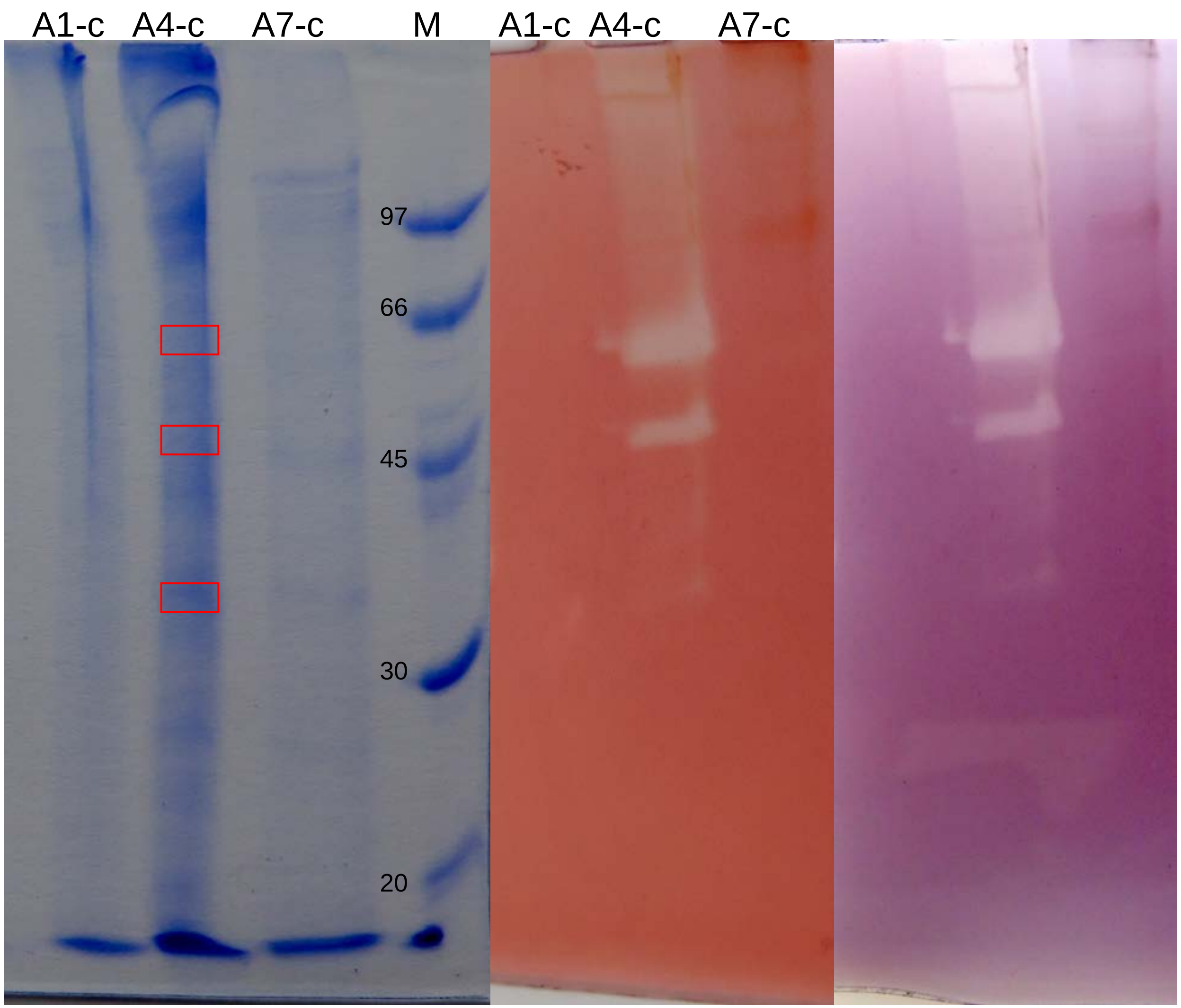

Supplementary Fig.S8. Endocellulases in denaturing PAGE (10%, 0.1% CMC). Proteins were denatured at 46°C for 2 h in Tris-HCl, pH 8 with 3% SDS. Renaturation at 4°C by two 30 min wash in Tris-HCl, pH 8 with 25% 2-PrOH followed by two washes without 2-PrOH. The activity was developed in 12 h at 37°C in 2 M Na^{+} soda/NaCl buffer, pH 9.5.
A1-c=AArcel1, cell extract; **A4-c=AArcel4**, cell extract; **A7-c=AArcel7**
The activity at this conditions can only be seen in strain AArcel4: ~60, 48 and 36 kDa
